# Multi-Omics Integration Analysis Identifies Lipid Disorder of a Non-Alcoholic Fatty Liver Disease (NAFLD) Mouse Model Improved by Zexie–Baizhu Decoction

**DOI:** 10.3389/fphar.2022.858795

**Published:** 2022-06-20

**Authors:** Yuhan Cao, Jingying Shi, Luyao Song, Junjiu Xu, Henglei Lu, Jianhua Sun, Jinjun Hou, Jing Chen, Wanying Wu, Likun Gong

**Affiliations:** ^1^ State Key Laboratory of Drug Research, Shanghai Institute of Materia Medica, Chinese Academy of Sciences, Shanghai, China; ^2^ University of Chinese Academy of Sciences, Beijing, China; ^3^ National Engineering Research Center of TCM Standardization Technology, Shanghai Institute of Materia Medica, Chinese Academy of Sciences, Shanghai, China; ^4^ School of Chinese Materia Medica, Nanjing University of Chinese Medicine, Nanjing, China

**Keywords:** NAFLD, metabolomics, transcriptomics, lipid metabolism, traditional Chinese medicine

## Abstract

Non-alcoholic fatty liver disease (NAFLD) is an increasingly epidemic metabolic disease with complex pathogenesis. Multi-target therapy may be an effective strategy for NAFLD treatment, and traditional Chinese medicine (TCM) characterized by multi-ingredients and multi-targets has unique advantages in long-term clinical practice. Zexie–Baizhu (ZXBZ) decoction is a Chinese classical formula to treat body fluid disorders initially. Although many bioactive monomers from Zexie and Baizhu had been discovered to improve lipid disorders, limited research studies were focused on the aqueous decoction of ZXBZ, the original clinical formulation. In the current study, we identified 94% chemical composition of ZXBZ decoction and first discovered its hepaprotective effect in a gubra-amylin NASH (GAN) diet-induced NAFLD mouse model. Based on metabolomics and transcriptomics analyses, we speculated that lipid and glucose metabolisms might be regulated by ZXBZ decoction, which was further confirmed by improved dyslipidemia and hepatic steatosis in ZXBZ groups. Consistently with cross-omics analysis, we discovered ZXBZ decoction could influence two energy sensors, Sirt1 and AMPK, and subsequently affect related proteins involved in lipid biosynthesis, catabolism, and transport. In conclusion, ZXBZ decoction regulated energy sensors, consequently impeded lipogenesis, and promoted fatty acid oxidation (FAO) to alleviate lipid disorders and protect the liver in NAFLD models, which suggested ZXBZ decoction might be a promising treatment for NAFLD.

## Introduction

Non-alcoholic fatty liver disease (NAFLD) is an emerging global health problem, especially in economically developed areas (Stefan et al., 2019). However, there are few officially approved drugs for NAFLD due to the extremely complicated pathogenesis (Konerman et al., 2018; Mullard, 2020). The most widely accepted hypotheses of NAFLD are the “two-hit hypothesis” and “multiple parallel hits hypothesis” (Day and James, 1998; Tilg and Moschen, 2010), and the consensus of these assumptions is that excessive hepatic lipid accumulation forms the first hit for the development of NAFLD (Lomonaco et al., 2012; Hirsova et al., 2016). As reported, the excessive lipid accrual in the liver leads to lipotoxicity, subsequently causing many adverse changes in hepatocytes, such as endoplasmic reticulum (ER) stress (Fu et al., 2012), mitochondria and lysosomal dysfunction (Manne et al., 2018), and impaired autophagy (Li et al., 2008). In addition, lipotoxicity can exacerbate glucose dysmetabolism (Ahmed et al., 2021), inflammation, and intestinal microbiota dysfunction (Bastin and Andreelli, 2020). These risk factors become the second hits to NAFLD, based on the excessive hepatic lipid accumulation as the first hit. Therefore, improving lipid metabolism is a fundamental therapeutic strategy for NAFLD.

Because of the tangled network of glucose and lipid disorders causing the complicated pathogenesis, there were limitations that single-target drugs might end up in adverse effects and compensatory feedbacks from other pathways, resulting in the lack of safe and effective drugs for NAFLD. In this case, seeking multi-target drugs which improve NAFLD phenotypically has unique advantages. Nowadays, traditional Chinese medicine (TCM) has garnered more interest in offering plentiful candidates for NAFLD treatment, with its effectiveness proved in long-term clinic use. Not only many bioactive monomers, such as silymarin and berberine are under phase four clinical trials (Yan et al., 2020), but also numbers of classical formulas have been proven to alleviate NAFLD (Dai et al., 2021; Yang, Sun, Wang, Zhang, Zhang, Gao et al.). Zexie–Baizhu (ZXBZ) decoction is a classical traditional Chinese medicine formula, which was initially written in *Synopsis of Prescriptions of the Golden Chamber (Jingui Yaolue, AD. ∼ 220)* in the Han dynasty to treat metabolism disorders of the body fluid, especially in the liver or stomach (abdomen). ZXBZ decoction is prepared with water in a ratio of 5:2 of Zexie (*Alismatis Rhizoma*, the rhizome of *Alisma plantago-aquatica* subsp. *orientale* (Sam.) Sam.) and Baizhu (*Atractylodis macrocephalae Rhizoma*, the rhizome of *Atractylodes macrocephala* Koidz.). Many extracts and some bioactive monomers of zexie and baizhu have been discovered to ameliorate metabolic diseases, especially lipid metabolism disorders. For instance, alisol A/alisol B/alisol-2,3-C from Zexie and atractylenolides (I, II, and III) from Baizhu can improve lipid metabolism via activating PI3K/Akt, AMPK, and JAK-STAT pathways (Chen et al., 2020; Wang et al., 2020; Yu et al., 2020; Sun et al., 2021a; Deng et al., 2021; Luan et al., 2021). Several kinds of alcohol extracts of zexie were also reported to benefit the lipid metabolism and relieve inflammation (Park et al., 2014; Jang et al., 2015; Zhang et al., 2017; Liu et al., 2019), and several TCM formulas containing Zexie and Baizhu are effective for NAFLD in previous studies (Fang et al., 2017; Feng et al., 2019a; Cheng et al., 2019; Tang et al., 2020). However, limited studies were performed on ZXBZ aqueous decoction itself. Since ZXBZ decoction is the most original prescription with effects proven by wide clinical applications since ancient China, we are determined to verify its efficacy in the NAFLD mouse model and investigate the underlying mechanisms for the further development and exploration of ZXBZ decoction.

In the current study, we first discovered the protective effects of ZXBZ decoction on the liver in the gubra-amylin NASH (GAN) diet–induced NAFLD model. Then, the multi-omics integration analyses including metabolomics and transcriptomics revealed the potential mechanisms of the lipid metabolism improved by ZXBZ decoction. Finally, the related genes including Sirt1, AMPK, and their downstream genes were detected by WB, RT-PCR, and IF, in which we found that ZXBZ decoction could protect the liver and balance lipid disorders in the NAFLD model via influencing AMPK and Sirt1. In addition, we have also observed similar pharmacological actions *in vitro*. Altogether, these results shed a light on the effects and mechanisms of ZXBZ decoction and propose further development of TCM toward NAFLD treatment.

## Materials and Methods

### Preparation and Verification of ZXBZ Decoction

Zexie pieces (*Alismatis Rhizoma*, the rhizome of *Alisma plantago-aquatica* subsp. *orientale* (Sam.) Sam., origin from Fujian, China) was obtained from Kangmei Pharmaceutical Co., Ltd., while Baizhu pieces (*Atractylodis macrocephalae Rhizoma*, the rhizome of *Atractylodes macrocephala* Koidz., origin from Anhui, China) was purchased from Shanghai Leiyunshang Co., Ltd. Dr. JJ Hou had authorized the two herbs of ZXBZ decoction based on Chinese Pharmacopoeia (2020 edition, Volume I). According to the “Synopsis of Prescriptions of the Golden Chamber,” the ratio of Zexie to Baizhu is 5:2 (w/w). Thus, zexie pieces (600.0 g) and Baizhu pieces (240.0 g) in ZXBZ decoction were socked with water (16.0 L) for half an hour. Then, they were slightly boiled for 2 h. The filtrates were concentrated by reducing pressure at 45°C, and freeze-dried into the water extract, in which the yield was 30.0%. The powder of water extract was stored at −20°C. Before gavage administration in mice, the water extract (75 and 150 mg/ml) was dissolved in 0.5% sodium carboxymethyl cellulose (CMC-Na).

### Animal Handling and Grouping

Six weeks old male C57/BL6 mice were purchased from Shanghai Laboratory Animal Co. (Shanghai, China) and fed in specific pathogen-free (SPF)-grade according to requirements of the Institutional Ethics Committee of Shanghai Institute of Materia Medica. The relative humidity was 30%–70%, the light/dark cycle was 12/12 h, and diet and drinking water were provided ad libitum. After 1-week adaptation and a 5-week gubra-amylin NASH (GAN) diet induction (rodent diets with 40 kcal% fat) (Primex or Palm Oil, Research Diets, United States) (Boland et al., 2019), the mice were distributed evenly into four groups according to body weights and ALT, besides normal control diet (NOD) mice were fed with regular diet and water (*n* = 10). Then the four GAN diet–induced groups were given vehicle (i.g.), obeticholic acid (30 mg/kg, i.g.), and ZXBZ decoction (750 and 1,500 mg/kg, i.g.) every day, respectively. After a feeding period of 17 weeks along with drug treatment of 12 weeks, the mice were sacrificed, and the liver and serum were collected for subsequent analysis.

### Serum Biochemistry Analysis

Blood was collected every 4 weeks via the tail veins of mice since drug or vehicle treatment began when mice had been fed on GAN diet for 5 weeks. The levels of serum ALT, AST, TC, TG, and LDL-C were measured using an automatic Roche biochemical analyzer with Roche kits (ALT, AST, TC, TG, and LDL-C).

### Fasting Blood Glucose, Oral Glucose Tolerance Test, Insulin Tolerance Test, and HOMA-IR

Fasting blood glucoses were determined after 6 hours of fasting using the OneTouch Select Simple^®^ glucose meter (Johnson & Johnson, United States). As for the oral glucose tolerance test (OGTT) and insulin tolerance test (ITT), the mice were fasted for 16 and 6 h, respectively, at the 11th and 13th week of GAN diet induction with the treatment of 1 g/kg glucose (i.g.) or 0.75 U/kg insulin (i.p.), . The glucose baseline levels were measured at 0, 15, 30, 45, 60, 90, and 120 min and calculated by the area under the curve (AUC). The homeostasis model assessment of the insulin resistance (HOMA-IR) level was performed at the 12th week of GAN diet induction and evaluated according to the formula HOMA-IR = [fasting plasma glucose (mmol/L) fasting plasma insulin (ng/ml)]/22.5. The insulin levels were examined using the ELISA kit (Beijing Solarbio Science & Technology Co., Ltd., Beijing, China).

### Histopathological Analysis

The livers were removed after a 12-week drug or vehicle treatment when HFD induction lasted for 17 weeks and fixed in 10% neutral buffered formalin to dehydrate for 3 days and embedded in paraffin. Serial transverse sections of 3–4 μm were stained with hematoxylin and eosin. To evaluate the degree of NAFLD quantificationally, the liver lesions were observed under low-power microscopy and examined following the NAFLD activity score (Kleiner et al., 2005).

The left lobes of livers were embedded by optimal cutting temperature compound (OCT) and frozen in liquid nitrogen immediately, then, the frozen tissues were cut into lesions and laid flat on glass slides. After staying at room temperature for 5 min, the samples were washed with distilled water and 60% isopropanol twice. Then, the samples were dyed with oil red O working solution for 5 min and terminated with distilled water and redyed with hematoxylin. The samples were observed and screened under a low-power microscope.

### Untargeted Urine Metabolomics Analysis

At 1, 5, and 9 weeks, urine was collected over 24 h through metabolic cages. The acquired urine samples were stored at −80°C pending sample preparation (Khamis et al., 2017; Feng et al., 2019b). The urine samples were thawed at room temperature before the measurement. The supernatant was diluted with water. The dilution factor was determined by the creatinine level measured in the urine sample by the HPLC procedure according to the Ministry of Health of the People’s Republic of China (People’s Republic of China, 1996). The diluted samples were centrifuged at 14,000 rpm for 10 min at 4°C for the UPLC–MS analysis. The exact UPLC–MS method has been reported before (Feng et al., 2019b). In brief, an LTQ-Orbitrap Velos Pro hybrid mass spectrometer (Thermo Fisher Scientific Corp.) linked to an Ultimate 3000 UHPLC system was used to produce high-resolution mass spectra for metabolomic investigation. A Waters ACQUITY UPLC HSS T3 column (1.8 μm, 2.1 mm × 100 mm) with an online filter was used to separate the samples. The mobile phase consisted of solvent A [100% H_2_O (0.1% formic acid)] and solvent B [100% acetonitrile (0.1% formic acid). The elution procedure is as follows: 0–1 min, 99% A; 1–3 min, 99%–85% A; 3–6 min, 85%–50% A; 6–9 min, 50%–5% A; and 9–10 min, 5% A. The flow rate remained constant at 0.5 ml/min. The autosampler and column were held at 4 and 40°C, respectively. The injection volume was fixed at 5 µl for all samples. The quality control (QC) sample was made up of an equal volume (10 μl) of each urine sample, and used to evaluate the peak intensity stability of metabolomics analysis. The QC sample was injected each after five samples.

### *In Vitro* Assay

HepG2 cells were plated in 6-well plates at a density of 1 × 10^5^ cells/ml and incubated in a humidified incubator at 37°C with 5% CO_2_. After the confluence reached 80%–90%, the culture medium was changed to the serum-free medium and the cells were starved for 12 h before treatment. Then, the medium was replaced by 1 mM oleic acid (OA)-palmitic acid (PA) = 2:1 or 500 μM PA medium. The ZXBZ decoction was filtered through a 0.22 μm membrane, and diluted into different concentrations. The negative control was 0.2% BSA and (0.1% DMSO or 0.1% ddH_2_O) containing medium, and the 10 μM Compound C and EX-527 were used to pre-treat the cells 1 h before ZXBZ intervention. After being co-incubated under different conditions for 24 h, the cells were lysed using 1% TritonX-100 or RIPA with 1% PMSF for the TG content assay or WB experiment, respectively.

### RNA Isolation and qRT-PCR Analysis of mRNA Expression

Total RNA was isolated using the TRIzol reagent (Yeasen Biotechnology (Shanghai) Co., Ltd.) and a UNlQ10 RNA extraction column kit (Sangon Biotech, Shanghai, China). cDNA was reverse transcribed using the PrimeScript™ RT Master Mix (Takara, Shiga, Japan), and qRT-PCR was analyzed on an ABI 7500 Fast system (ABI, CA, United States) using the Hieff^®^ qPCR SYBR Green Master Mix (Yeasen, Shanghai, China). All results were normalized to the RPS18 expression and calculated using the 2−(ΔΔCt) method and the primers of qRT-PCT are provided in Table 1.

**TABLE 1:** Primers for qRT-PCR.

Primer	Sequence
RPS18 forward primer	5′-CGC​CGC​CAT​GTC​TCT​AGT-3′
RPS18 reverse primer	5′-CCC​TCT​TGG​TGA​GGT​CGA​TG-3′
SREBP-1c forward primer	5′-CTG​CTA​GCT​AGA​TGA​CCC​TGC-3′
SREBP-1c reverse primer	5′-TCT​GGC​TTT​GAT​CCC​GGA​AG-3′
CHREBP forward primer	5′-CTG​GGG​ACC​TAA​ACA​GGA​GC-3′
CHREBP reverse primer	5′-GAA​GCC​ACC​CTA​TAG​CTC​CC-3′
CPT1A forward primer	5′-CAT​GTC​AAG​CCA​GAC​GAA​G-3′
CPT1A reverse primer	5′-TGG​TAG​GAG​AGC​AGC​ACC​T-3′
G6PC forward primer	5′-TTA​CCA​AGA​CTC​CCA​GGA​CTG-3′
G6PC reverse primer	5′-GAG​CTG​TTG​CTG​TAG​TAG​TCG-3′
CYP7A1 forward primer	5′-TGA​TCC​TCT​GGG​CAT​CTC​AAG​CAA-3′
CYP7A1 reverse primer	5′-AGC​TCT​TGG​CCA​GCA​CTC​TGT​AAT-3′
CYP27A1 forward primer	5′-TTG​CCT​GGA​TAG​GGC​TCA​TAG-3′
CYP27A1 reverse primer	5′-GTG​GGG​CAC​TAG​CCA​GAT​TC-3′
LXRA forward primer	5′-GCC​CTG​CAC​GCC​TAC​GT-3′
LXRA reverse primer	5′-TAG​CAT​CCG​TGG​GAA​CAT​CA-3′
Creb-forward primer	5′-GAC​GGA​GGT​TAA​GTC​GAG​CC-3′
Creb-reverse primer	5′-TCT​TCC​TCC​GCA​CTC​GTT​TC-3′
LPL forward primer	5′-CCA​GCT​GGG​CCT​AAC​TTT​GA-3′
LPL reverse primer	5′-AAC​TCA​GGC​AGA​GCC​CTT​TC-3′
HMGCR forward primer	5′-GCT​ACT​GGG​ATG​GTC​GCT​AT-3′
HMGCR feverse primer	5′-TTG​AAC​ATG​TCC​AGG​GAG​GC-3′

### Western Blotting

Mouse livers (20 mg) were lysed using RIPA lysis buffer (Beyotime, China) containing 1% cock-tail (Sigma-Aldrich, St Louis, MO, United States). Total protein lysates were separated on 10% SDS-PAGE gels and then transferred to PVDF membranes (Millipore, United States), and then were incubated overnight at 4°C with antibodies against sirtuin 1 (Sirt1), histone 3, phospho-AMPKα (Thr172), and AMPKα (Cell Signaling Technology, United States), PGC-1α, phospho-ACC1-S79, ACC, phospho-mTOR-S2448, SREBP-1c, PPARα (ABclonal Technology Co., Ltd., Wuhan, China), β-actin, and β-tubulin (Abcam, MA, United States). The membranes were washed and incubated with HRP-conjugated secondary antibodies (Jackson ImmunoResearch Laboratories, Inc., United States) and then detected using an ECL Plus immunoblot detection system (Clinx, Shanghai, China).

### Data Processing and Statistical Analysis

SIMCA-P software (version 14.1, Umetrics AB) was employed for multivariate analysis. Principal component analysis (PCA) analysis was carried out to demonstrate the aggregation of QC samples. Bidirectional orthogonal projection to latent structures discriminant analysis (O2PLS-DA) analysis was performed to describe the different metabolic profiles of the NC, vehicle, ZXBZ-L, ZXBZ-H, and positive groups. Orthogonal partial least squares discriminant analysis (OPLS-DA) analysis was used to better investigate the metabolic difference between the NC group and the vehicle group. R2 and Q2 were calculated to evaluate the quality of the model, and 200 permutation tests were performed to test the model. Potential biomarkers were confirmed based on the *p* < 0.05 and VIP > 1 between the NC and vehicle groups.

The potential urine biomarkers were identified using the metabolite database HMDB (http://www.hmdb.ca/) based on the MS^1^ and MS^2^ information. The pathway analysis was performed using MetaboAnalyst (http://www.metaboanalyst.ca/).

GSEA analysis was performed on GSEA 4.2.0 (36, 37) using the Reactome database (https://reactome.org/). The results were visualized using the R code (version 4.0.2) developed by us in RStudio. Paths were sorted by NES (NES < −1 or NES > 1 was considered significantly enriched).

The data from animal experiments are presented as the mean ± SEM, and *in vitro* experimental data are presented as the mean ± SD. Student’s *t*-tests were used to compare two groups. Comparisons among multiple groups were made with a one-way analysis of variance (ANOVA). A value of *p* < 0.05 was considered statistically significant.

## Results

### Components of ZXBZ Decoction Are Identified by Multiple Analytical Methods

**FIGURE 1:**
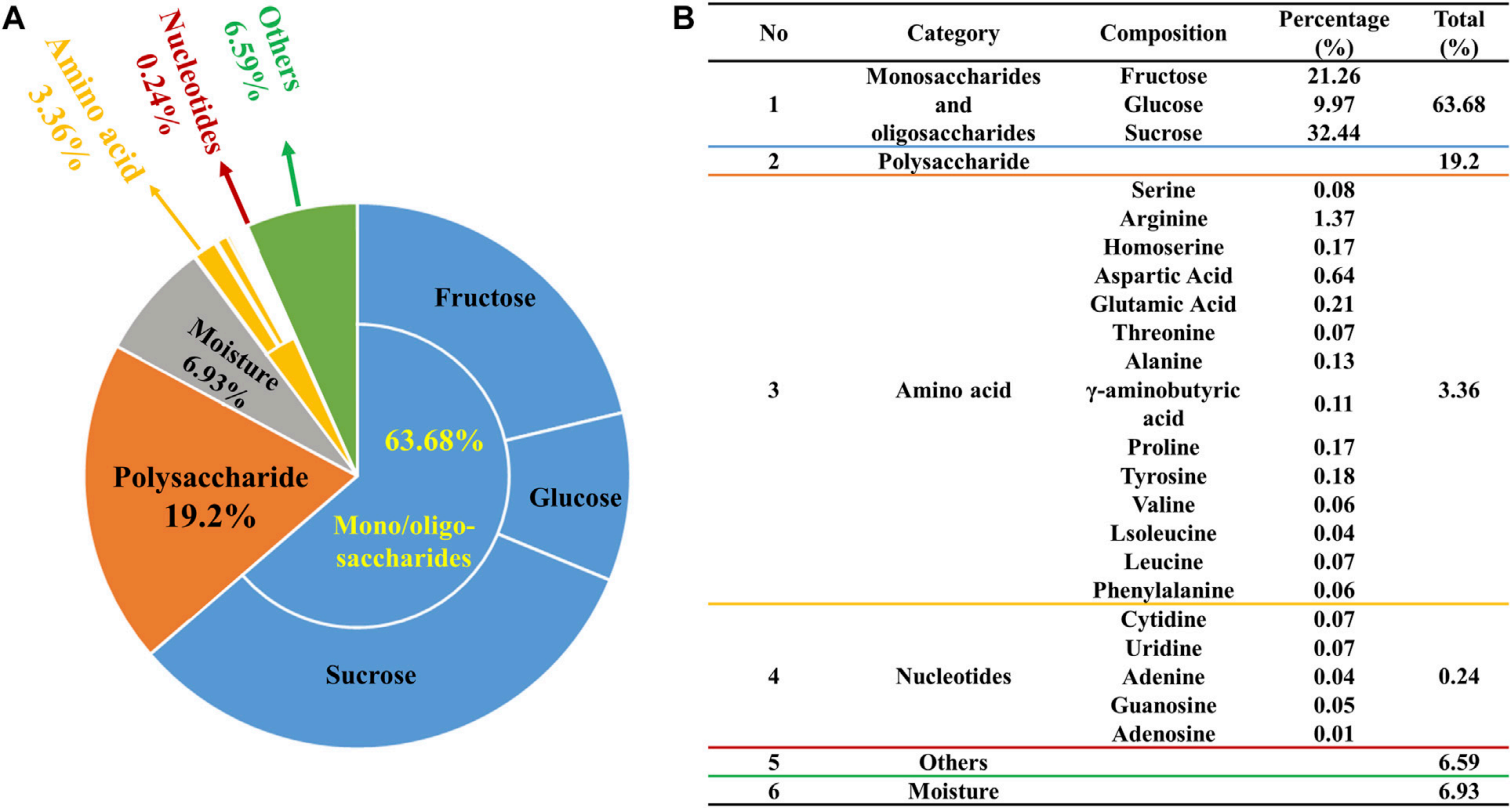
Quantitative characterization of ZXBZ decoction. Distribution of **(A)** the general and **(B)** specific chemical composition of ZXBZ decoction.

The 94% chemical composition of ZXBZ decoction (Batch 20191129) was identified with four assays (Figure 1A). First, the contents of monosaccharides and oligosaccharides were determined using the high-performance liquid chromatography-diode array detection (HPLC-DAD) method (Supplementary Material 1), in which fructose accounted for 21.26%, glucose for 9.97%, and sucrose for 32.44%. The total content of the three saccharides had reached 63.7% of the aqueous extracts. Second, the contents of polysaccharides were analyzed using the phenol–sulfuric acid method (Chen et al., 2019), in which their percentage in the aqueous extract composition was 19.2%. Third, the varieties and contents of amino acids were assayed using the AccQ•Tag pre-column derivation method (Boogers et al., 2008). Fourteen amino acids were detected at a total content of 3.36%, among which the highest amino acid was arginine (1.37%). Fourth, a total of five nucleotides were quantified (Supplementary Material 2) using the HPLC-UV method and their total content was 0.24%. At last, the water content of ZXBZ aqueous extracts was determined using USP 40 <921> method III (rtf), in which the result was 6.93% (Figure 1B). The representative total ion chromatogram of ZXBZ was also characterized (Supplementary Figure S1) and 33 compounds were identified (Supplementary Table S1).

### ZXBZ Decoction Impedes Liver Injury in the GAN Diet-Induced NAFLD Model

**FIGURE 2:**
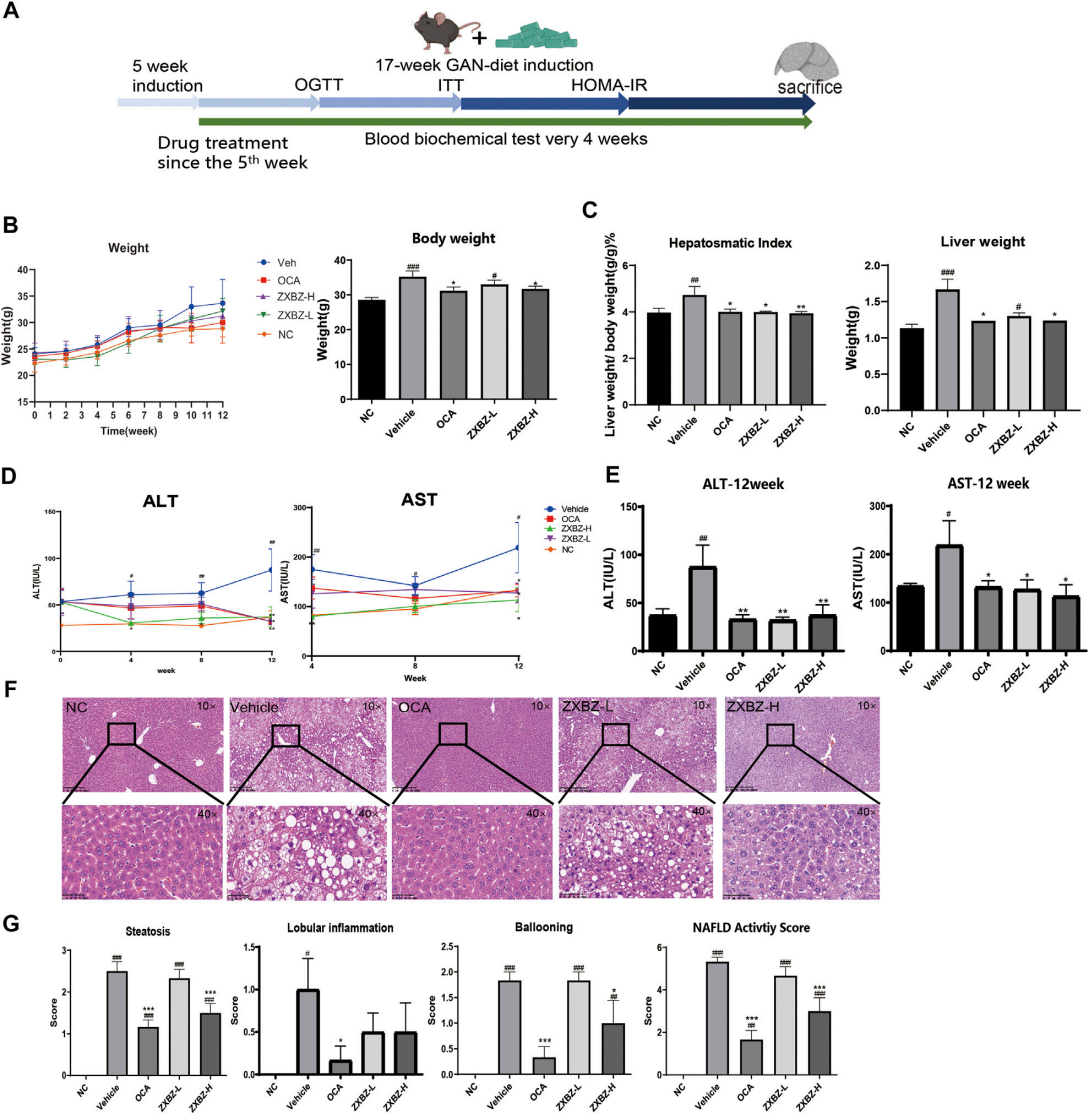
Liver protective effect of ZXBZ decoction. **(A)** Design and timeline of animal experiments. **(B)** Body weight throughout the whole drug treatment process and the end point body weight. **(C)** Hepatosomatic index and the liver weight. **(D)** ALT and AST in the drug treatment process. **(E)** End point ALT and AST. **(F)** H&E staining of each group (10× and 40×). **(G)** NAFLD activity score. The histological NAS scores of necroinflammatory, ballooning, and macrovesicular steatosis were determined by a certified pathologist. The data are the mean ± SEM (*n* = 8–10 per group). #*p* < 0.05, ##*p* < 0.01, ###*p* < 0.001 compared to NC. *p < 0.05, **p < 0.01, ***p < 0.001 compared to vehicle.

Given the regulatory role of ZXBZ decoction as a classical formula in the hepatic fluid metabolism disorder, we wondered whether it can modulate other metabolic pathways of the liver, especially glucose and lipid metabolism. Thus, the GAN diet–induced NAFLD mouse model was used, where C57 BL/6 mice were pre-fed with the GAN diet for 5 weeks and administered ZXBZ decoction by oral gavage daily for 12 weeks (Figure 2A). The GAN diet is a commonly used diet to induce NAFLD/NASH models, which shares similar characteristics with NAFLD/NASH patients in the respects of histopathology, transcription, and metabolism (Hansen et al., 2020; Radhakrishnan et al., 2020). In this experiment, we selected obeticholic acid (OCA), a candidate for NAFLD treatment in phase III clinical trials (Ratziu et al., 2016), as the positive control for the therapeutic effects. As shown in Figure 2B, the body weights of the vehicle group increased gradually with GAN diet feeding when compared with that of the NC group fed by the normal diet, while the body weights were obviously lowered no matter in the positive group or the low and high dosages of ZXBZ treated (ZXBZ-L and ZXBZ-H) groups than those in the vehicle group. Moreover, the hepatosomatic indexes of OCA, ZXBZ-L, and ZXBZ-H groups and liver weights of OCA and ZXBZ high groups were also markedly lower than vehicles after 12 weeks of continuous administration (Figure 2C).

To assess the effects of ZXBZ decoction on the liver function, the serum biomarkers, aspartate aminotransferase (AST), and alanine aminotransferase (ALT), were detected. Compared to the vehicle group, the ZXBZ-L and ZXBZ-H groups both exhibited lower ALT and AST levels since the fourth week of drug treatment, which was continued until the end of the experiment (Figures 2D,E). Afterward, the pathological changes in liver tissues from different groups were observed by H&E staining and quantified by NAFLD activity score (NAS). The vehicle group with a NAS of 5 had significantly hepatic steatosis, ballooning, and lobular inflammation compared to NC and reached the threshold for the diagnosis of NASH (Brunt et al., 2011). The high dosage ZXBZ treatment could alleviate hepatic steatosis and ballooning of hepatocytes obviously and relieved the lobular inflammation slightly without significance (Figures 2F,G).

The aforementioned results showed that ZXBZ decoction could reduce the levels of ALT and AST, relieve hepatic steatosis, and ballooning degeneration significantly in the GAN diet–induced NAFLD model.

### Metabolomics Reveals That ZXBZ Decoction Could Improve the β-Oxidation

To monitor the metabolic changes, the urine metabolites were collected monthly and studied. The PCA score plot showed good stability and feasibility of the detection method due to the aggregation of points in the QC group (Supplementary Figure S2A). As illustrated by the O2PLS-DA score plot (Supplementary Figure S2B), there was an excellent separation among the NC and vehicle groups, indicating that the urine metabolites in NAFLD mice were significantly changed. The ZXBZ-H and ZXBZ-L groups were close to each other and had similar metabolic phenotypes. In addition, the ZXBZ-H group could be separated from the vehicle group, showing the benefits of ZXBZ at the metabolic level.

**FIGURE 3:**
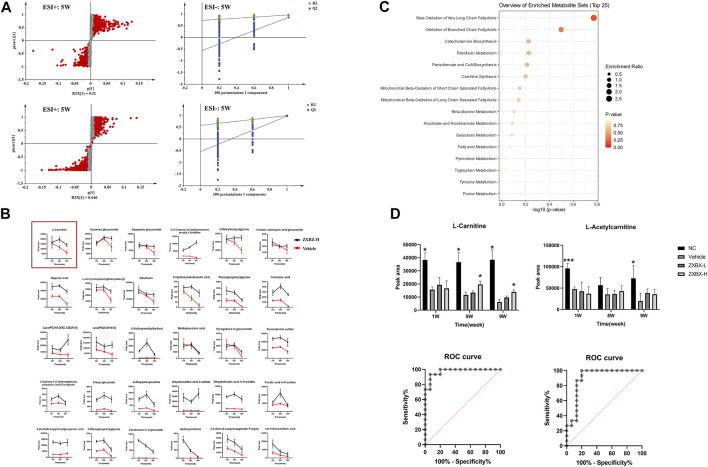
Metabolomics revealed that ZXBZ decoction could improve the β-oxidation. **(A)** OPLS-DA s-plot and permutations of urine data in the positive mode and negative mode at fifth week. **(B)** Relative intensities of 30 metabolites stably present in three samples. **(C)** Summary of pathway analysis of urine samples. **(D)** Relative intensities and receiver operating characteristic (ROC) for L-carnitine and L-acetylcarnitine.

An OPLS-DA model was established to search for potential biomarkers of NAFLD between the NC and vehicle groups (Figure 3A and Supplementary Figures S2C,D). In addition, the data were categorized according to different time points. The parameters of the model (the R2Y and Q2 values should be near to 1, indicating a good ability to forecast) were acceptable, indicating good prediction and reliability. Furthermore, the models were validated using permutation tests (*n* = 200). Variables with VIP values > 1 and *p* values < 0.05 were identified as significant endogenous biomarkers. The candidate ions were tentatively identified by searching the MS and MS/MS fragments using the online library (http://www.hmdb.ca/). Finally, 60 metabolites were identified as potential biomarkers in the NAFLD model. The results of the detailed identification information and shifting trends of biomarkers are shown in Supplementary Table S1. Overall, 30 metabolites were stably present in all three sampling occasions, and the reversal effect was reflected in the ZXBZ administration groups (Figure 3B and Supplementary Figure S2E).

To further investigate the metabolic pathways disturbed in NAFLD, an online tool, MetaboAnalyst 5.0 software (www.metaboanalyst.ca/), was used and 16 related pathways were found to be affected (Figure 3C). Among them, beta-oxidation of very long-chain fatty acids and oxidation of branched-chain fatty acids were the two most important pathways in the urine metabolite pathways, including L-carnitine and L-acetylcarnitine. As shown in Figure 3D, L-carnitine and L-acetylcarnitine decreased in the vehicle group and increased after ZXBZ treatment. Moreover, the AUC of the two metabolites were 0.9733 and 0.8978, both sensitive to being a biomarker, especially L-carnitine. Moreover, L-carnitine and L-acetylcarnitine were identified using available standards (Supplementary Figure S3).

### Transcriptomics Reveals That ZXBZ Decoction Could Improve Energy Metabolism

**FIGURE 4:**
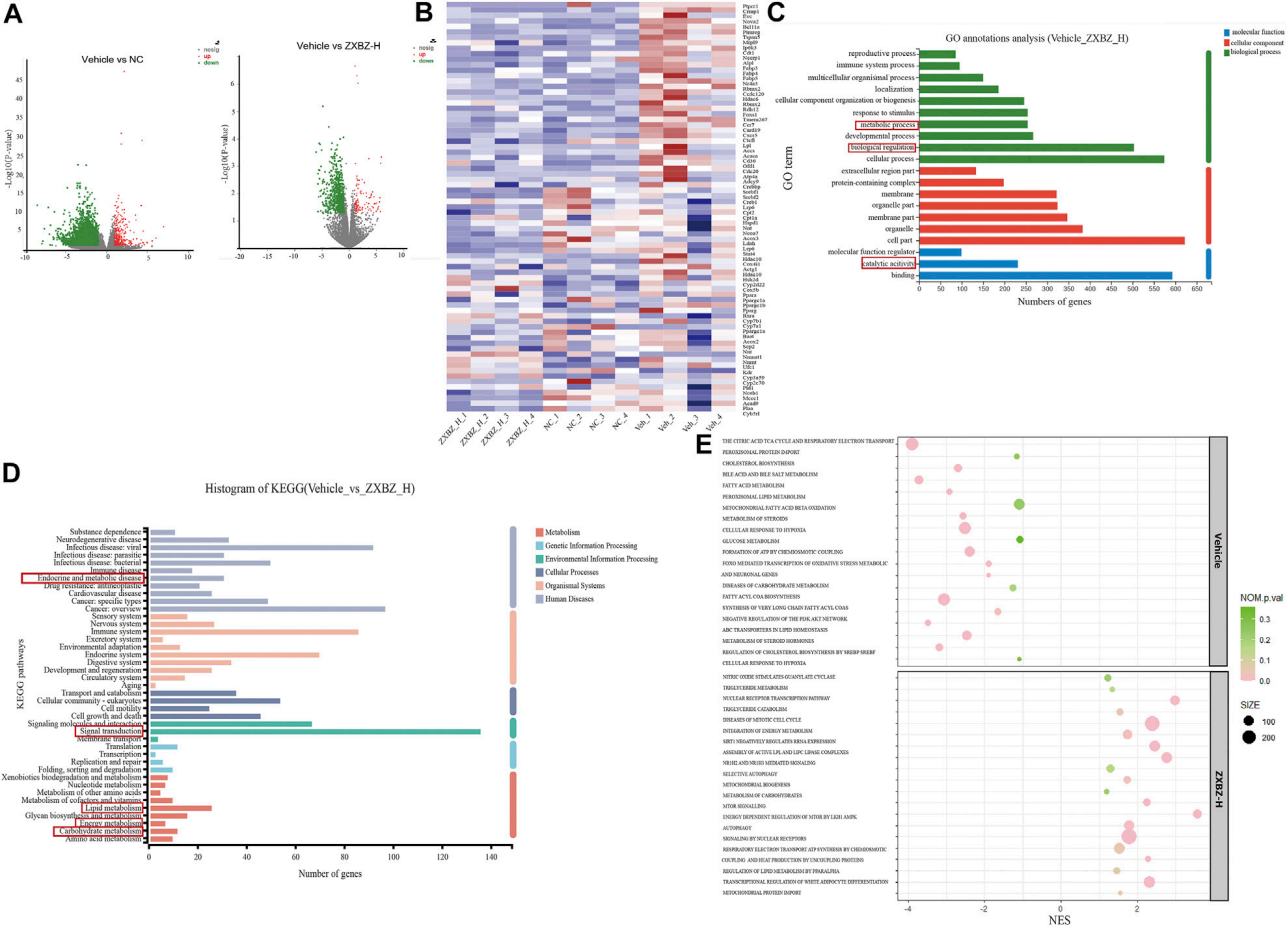
RNA sequencing of liver tissue indicated ZXBZ decoction prevented liver injury via improving energy metabolism. **(A)** Volcano plots of NC or ZXBZ-H vs. vehicle. **(B)** Heatmap of different expression genes. **(C)** GO annotations of significantly differentially expressed genes (SDEGs) from ZXBZ-H group vs. vehicle. **(D)** KEGG annotations of SDEGs from ZXBZ-H group vs. vehicle. **(E)** GSEA by the Reactome pathway database.

To study the underlying mechanisms of ZXBZ’s hepatoprotective functions thoroughly, high-throughput RNA-seq technology was used to profile liver transcriptomes of vehicle, NC, and ZXBZ-H groups, respectively (*n* = 4/group), and the data were analyzed on the free online platform of the Majorbio Cloud Platform (http://www.majorbio.com/) and GSEA (https://www.gsea-msigdb.org/gsea/). The overall significantly differentially expressed genes (SDEGs) in NC and ZXBZ-H groups compared to the vehicle group were shown in the volcano chart (Figure 4A). The details of SDEGs related to NAFLD were shown in the cluster heatmap (Figure 4B), including the genes of lipid metabolism and transport such as *Lpl*, *Accs*, and *Cd36*, nuclear receptors such as *Ppara*, *Pparg*, and *Rxra*, electron transport chain, for instance, *Atp4*, *Cox4*, and *Nmant*, and bile acid metabolism-related genes, such as *Cyp7a1*. The SDEGs between ZXBZ-H and vehicle groups were further analyzed KEGG pathway and GO annotation, respectively, and the results demonstrated that ZXBZ decoction mainly regulated lipid metabolism, glycan biosynthesis and metabolism, and carbohydrate metabolism (Figures 4C,D). The gene set enrichment analysis (GSEA) was also performed to reveal the potential pathways and biological processes influenced by ZXBZ decoction (Figure 4E), and the results indicated that ZXBZ decoction might balance cellular energy states. The lipogenesis (FAs, cholesterol, and sterols) and hypoxia were enhanced in the vehicle group, whereas the genes involved in the mitochondrial function including ATP synthesis and mitochondrial biogenesis, nitric oxide metabolism, and nuclear receptor (PPAR and LXR) pathways were enriched in the ZXBZ-H group. Interestingly, these pathways are regulated by Sirt1. In addition, autophagy and mTOR pathways indicated the activation of the AMPK/mTOR pathway.

The transcriptomic data suggested that ZXBZ decoction improved energy metabolism, especially lipid metabolism, by affecting energy sensing and regulatory pathways.

### ZXBZ Decoction Alleviates Hepatic Steatosis and Dyslipidemia

**FIGURE 5:**
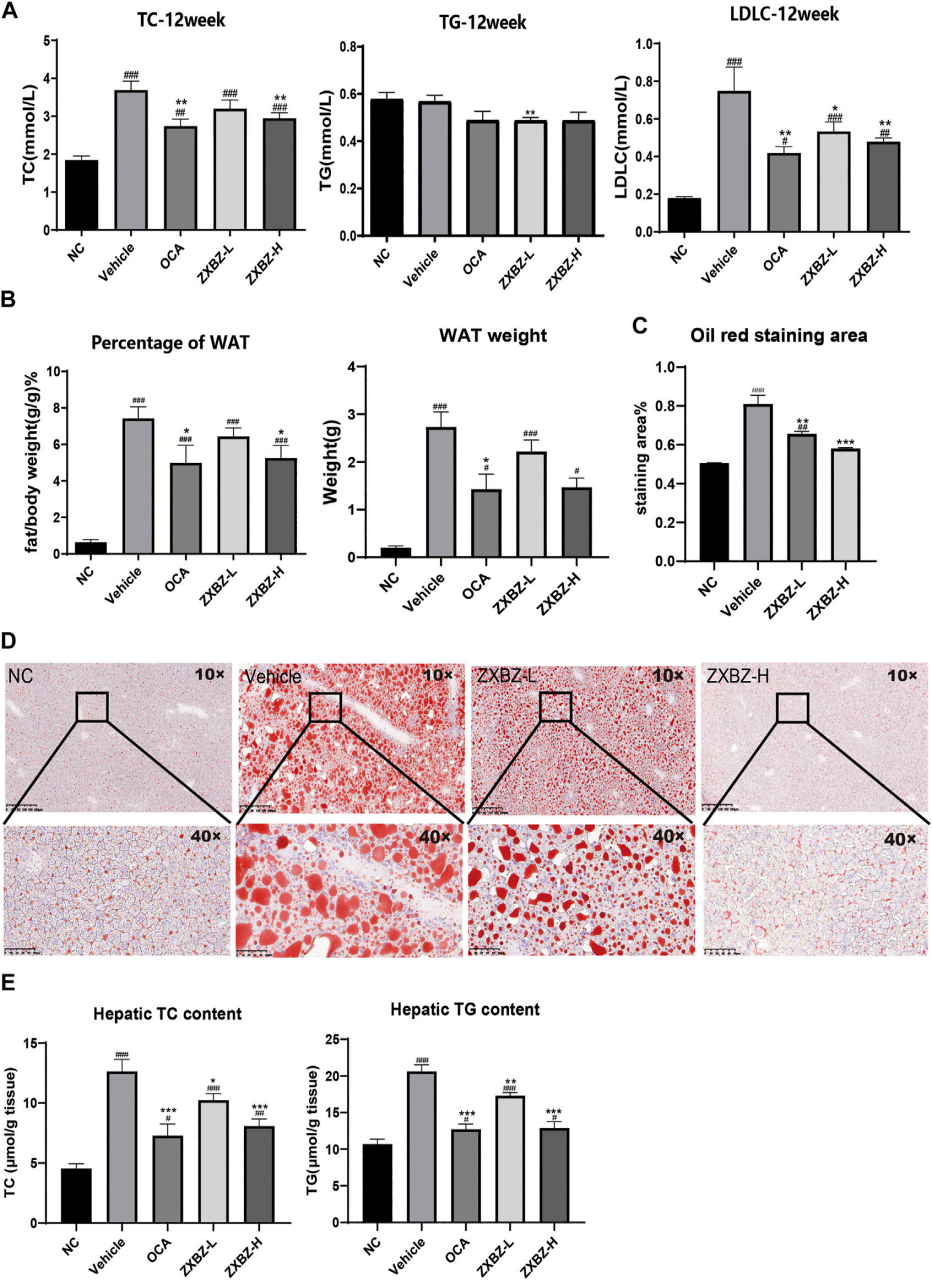
ZXBZ decoction could improve the lipid metabolism in NAFLD mice. **(A)** Levels of TC, TG, and LDLC in serum after 12-week treatment. **(B)** Percentage of white adipose tissue and white adipose tissue weight of each group. **(C)** The oil red staining area of liver tissues. The positive areas were calculated using Image Pro plus 6.0. **(D)** Oil red O staining of livers and quantification in NC, vehicle, ZXBZ-L, and ZXBZ-H groups. **(E)** The TC and TG contents in mouse livers and normalized by tissue weight. The data are the mean ± SEM (*n* = 8–10 per group). #*p* < 0.05, ##*p* < 0.01, ###*p* < 0.001 compared to NC. *p < 0.05, **p < 0.01, ***p < 0.001 compared to vehicle.

Since the multi-omics results clued ZXBZ decoction might influence energy metabolism, particularly lipid metabolism, we assessed the lipid metabolism in NAFLD mice among different groups. The total cholesterol (TC) and low-density lipoprotein cholesterol (LDLC) in serum were elevated in response to the nutritious diet stimulation, while the high dose of ZXBZ could decrease the TC and LDLC (Figure 5A). The parallel results also appeared in the ratio of white adipose tissue (WAT)/bodyweight and WAT weights (Figure 5B). The oil red O staining (Figures 5C,D) and lower hepatic steatosis score (Figure 2E) demonstrated the reduced hepatic lipid accumulation and steatosis intuitively from histology. In addition, the cholesterol and triglycerides (TGs) in liver tissue were reduced in OCA and ZXBZ-L and H groups compared to vehicle group (Figure 5E).

Taken together, we can conclude that ZXBZ decoction could ameliorate lipid disorders in the aspects of reducing circulating lipid levels and hepatic steatosis.

### ZXBZ Decoction Regulates the Energy-Sensing Network to Improve Lipid Metabolism

**FIGURE 6:**
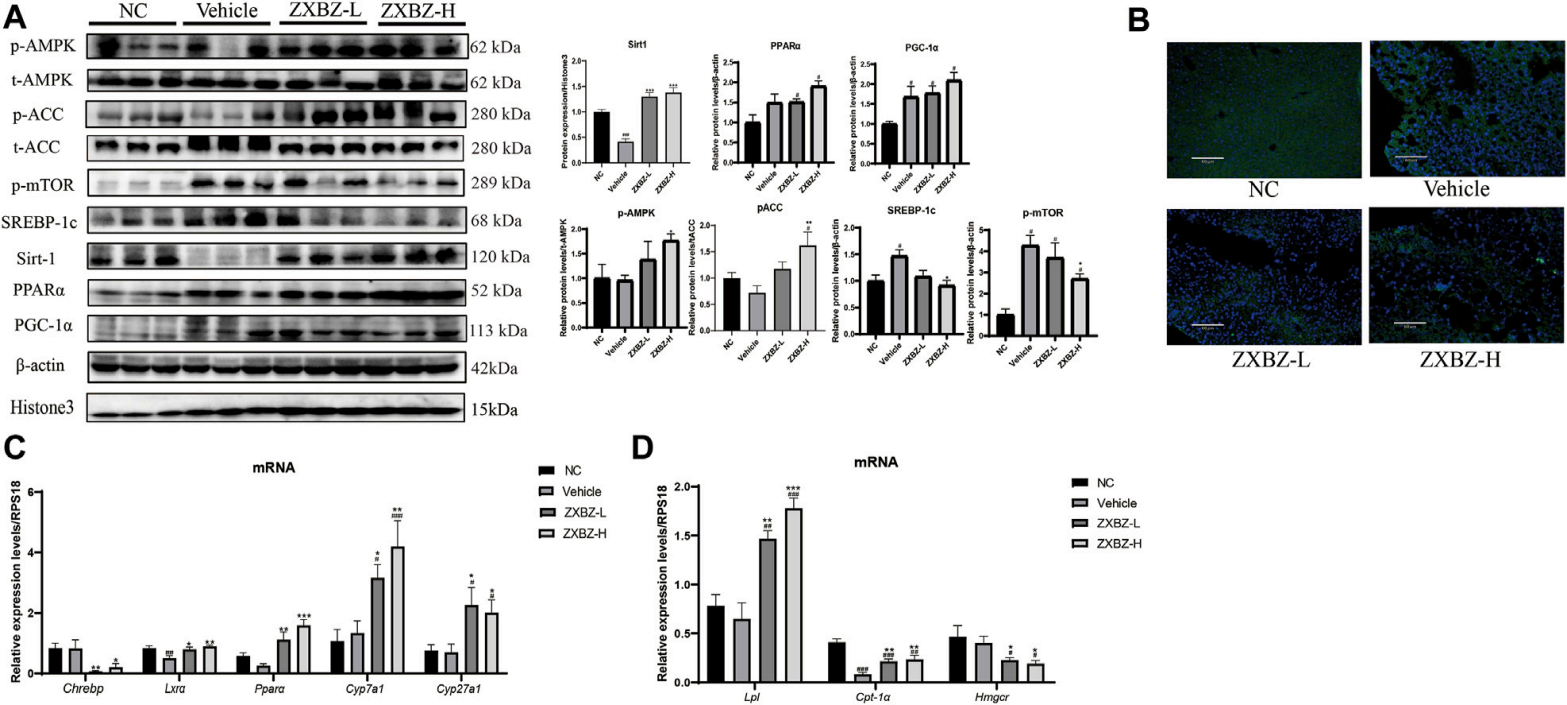
ZXBZ decoction could influence lipid metabolism–related proteins in Sirt1 and AMPK pathway. **(A)** Western blotting and related quantification of NC, vehicle, ZXBZ-L, and ZXBZ-H group. Data are mean ± SEM (*n* = 3 per group). **(B)** Immunofluorescence of FASN. Blue represented cell nucleus, and green represented FASN in the cytoplasm. **(C,D)** mRNA levels of genes determined by qRT-PCR. The data are the mean ± SEM (*n* = 6 per group). #*p* < 0.05, ##*p* < 0.01 compared to NC. *p < 0.05, **p < 0.01 compared to vehicle.

We further confirmed the possible molecular mechanisms based on the remarkable phenotypic improvements in the lipid disorder of NAFLD models and multi-omics analysis results. As shown in Figure 6A, both dosages of ZXBZ treatment increased Sirt1 and activated AMPK, two nutrient sensors responding to NAD^+^/NADH and AMP/ATP, respectively in mammalian cells (Fulco and Sartorelli, 2008; Wang et al., 2011). As reported, Sirt1 activation can suppress *de novo* lipogenesis by inhibiting SREBP-1c and *Chrebp*, and promote fatty acid oxidation (FAO) by increasing PPARα and PGC-1α expressions (Cantó and Auwerx, 2009). The corresponding changes in ZXBZ treatment on these genes were consistent with the previous research works. Similarly, the downstream genes of PPARα, such as *Cyp7a1*, C*yp27a1*, and *Lxrα* (Defour et al., 2012) were also increased under the condition of ZXBZ treatment, which indicated that ZXBZ might regulate bile acid metabolism, stimulate cholesterol clearance, and reverse cholesterol transport from peripheral tissues (Liang et al., 2021). Moreover, the activation of AMPK by ZXBZ consequently inactivated ACC and further reduced FASN (Figure 6B) and SREBP-1c, while increasing the *Cpt-1α* expression to reduce the fatty acid synthesis and promote the fully oxidation of long-chain and very long-chain FAs (Huang et al., 2017). We also found that AMPK activation downregulated *Hmgcr* transcription, meanwhile increasing *Lpl* mRNA levels (Figure 6C), which might reduce cholesterol synthesis and accelerate TG decomposition (Figure 6D) (Day et al., 2021; Trefts and Shaw, 2021). Furthermore, the phosphorylation of mTOR was inhibited in ZXBZ groups, suggesting autophagy was prompted after ZXBZ treatment, which could protect hepatocytes (Kim and Guan, 2021).

Altogether, ZXBZ decoction mainly regulated the energy-sensing network by influencing the Sirt1 expression and AMPK activation to govern lipid metabolism–related protein expressions and activations, consequently inhibiting lipogenesis and boosting lipids utilization in GAN diet–induced NAFLD mice.

### ZXBZ Decoction Has a Weak Effect on Glucose Metabolism in GAN Diet-Induced NAFLD Models

**FIGURE 7:**
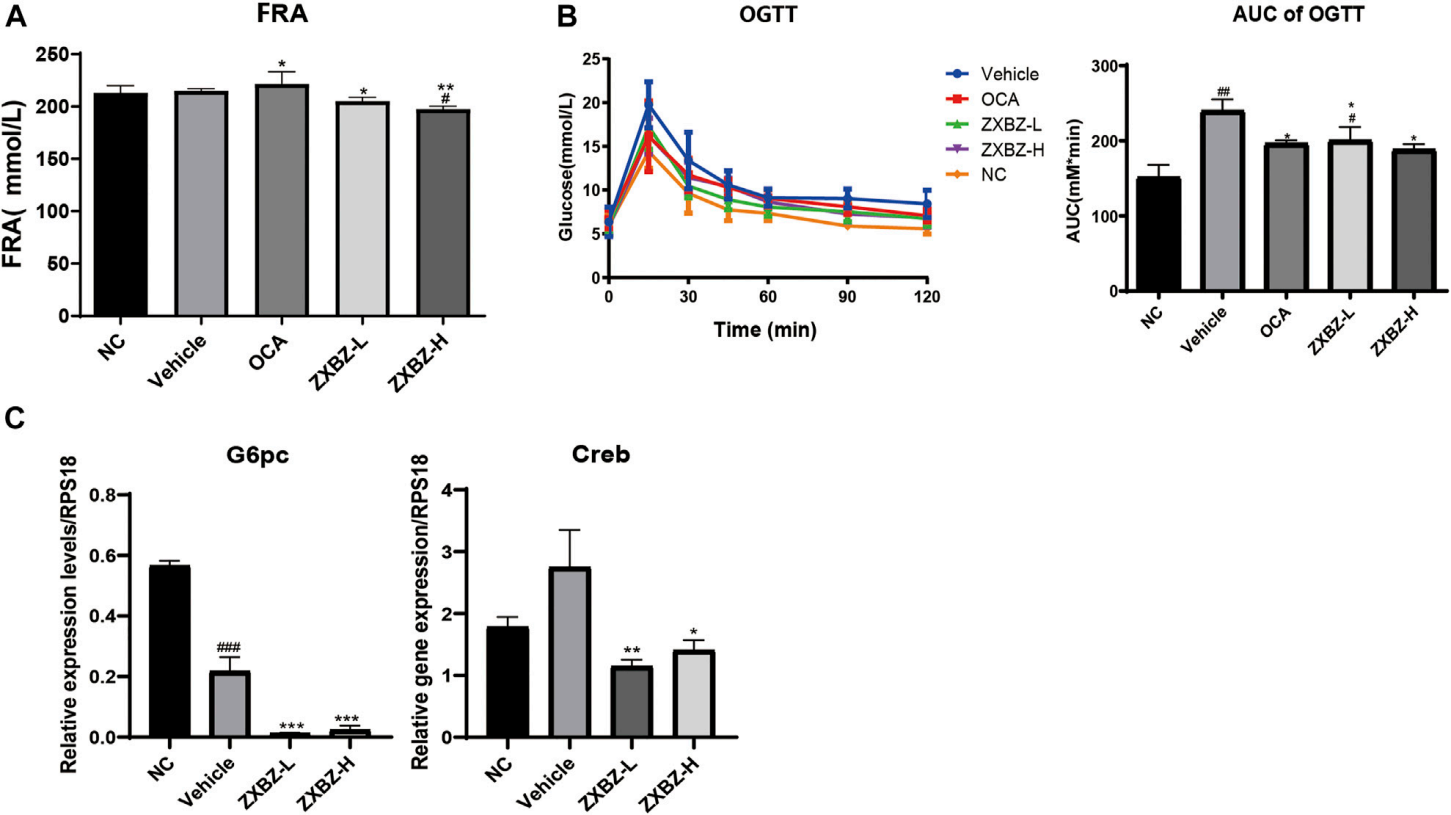
ZXBZ decoction improved glucose metabolism slightly. **(A)** Serum fructosamine at the end point. **(B)** Oral glucose tolerance test assay and related area under the curve. Data are shown in mean ± SEM (*n* = 8–10 per group). **(C)** mRNA levels of related genes detected by qRT-PCR #*p* < 0.05, ##*p* < 0.01 compared to NC. *p < 0.05, **p < 0.01 compared to vehicle.

Since glucose metabolism is another important assessment indicator in NAFLD, the oral glucose tolerance test (OGTT), HOMA-IR, and the insulin tolerance test (ITT) were measured on the sixth, seventh, and eighth week. As shown in Supplementary Figure S4, insulin resistance was not evident in this model, because there was no change in HOMA-IR and only an 18.65% increase in the AUC of the ITT between NC and vehicle groups. For OGTT with the obvious change between the NC and vehicle groups, we found ZXBZ decoction showed obvious improvement (Figure 7A). In addition, the serum fructosamine levels, which reflected blood sugar levels in the last one to 3 weeks, were also measured at the end of the experiment. Although serum fructosamine level had a slight change under diet induction, the administrated ZXBZ groups showed a weak but statistically significant reduced fructosamine level (Figure 7B). Meanwhile, the expression of gluconeogenesis genes *G6pc* and *Creb* which can be upregulated by AMPK activation were significantly hindered by ZXBZ treatment, which indicated that ZXBZ decoction may have regulatory effects in glucose metabolism (Figure 7C).

To sum up, the 12 weeks of ZXBZ administration improved the glucose tolerance, decreased serum fructosamine, and reduced gluconeogenesis gene transcriptions in the GAN diet–induced NAFLD mice model.

## Discussion

Nowadays, obesity-related diseases, especially NAFLD, have become a heavy burden for the global society, but the economical and effective treatments are still badly unmet. Most studies believe that lipid disorder is not only the primary risk factor for NAFLD development but a key trigger for other metabolic diseases as well. However, clinical applications suggested single-target drugs have many drawbacks, as a result, classical TCM formulas, with the advantages of multi-ingredients, multi-targets, and multi-pathways, have drawn increasing interest as potential treatments for NAFLD.

ZXBZ decoction was a classical TCM formula serving as a treatment for metabolism disorders in the body fluid since ancient time, and the alcohol extracts and monomers of Zexie and Baizhu, had been proven to improve lipid metabolism in modern studies. In this study, we proved that the chemical composition of ZXBZ decoction is dominated by polar glycoconjugates, especially polysaccharides, while the secondary metabolites are very low. This indicates that the presence of polysaccharide macromolecules in traditional Chinese medicine tonics cannot be ignored. The beneficial regulation of gut microbiota has been widely reported for polysaccharides, including providing energy for intestinal microorganisms, shaping the diversity of the gut microbiota, protecting bacteria from environmental, and host factors, including the host immune system, and so on (Porter and Martens, 2017; Hsieh and Allen, 2020; Sun et al., 2021b; Song et al., 2021). Thus, the polysaccharide in ZXBZ may be separated and tested further for understanding its pharmacological substances. Considering the ZXBZ decoction was the most original, convenient, and widely used formulation in folk, we aimed to verify the therapeutic effects of ZXBZ decoction in the NAFLD mice model and reveal the underlying mechanisms combining multi-omics analysis and molecular biological verification.

**FIGURE 8:**
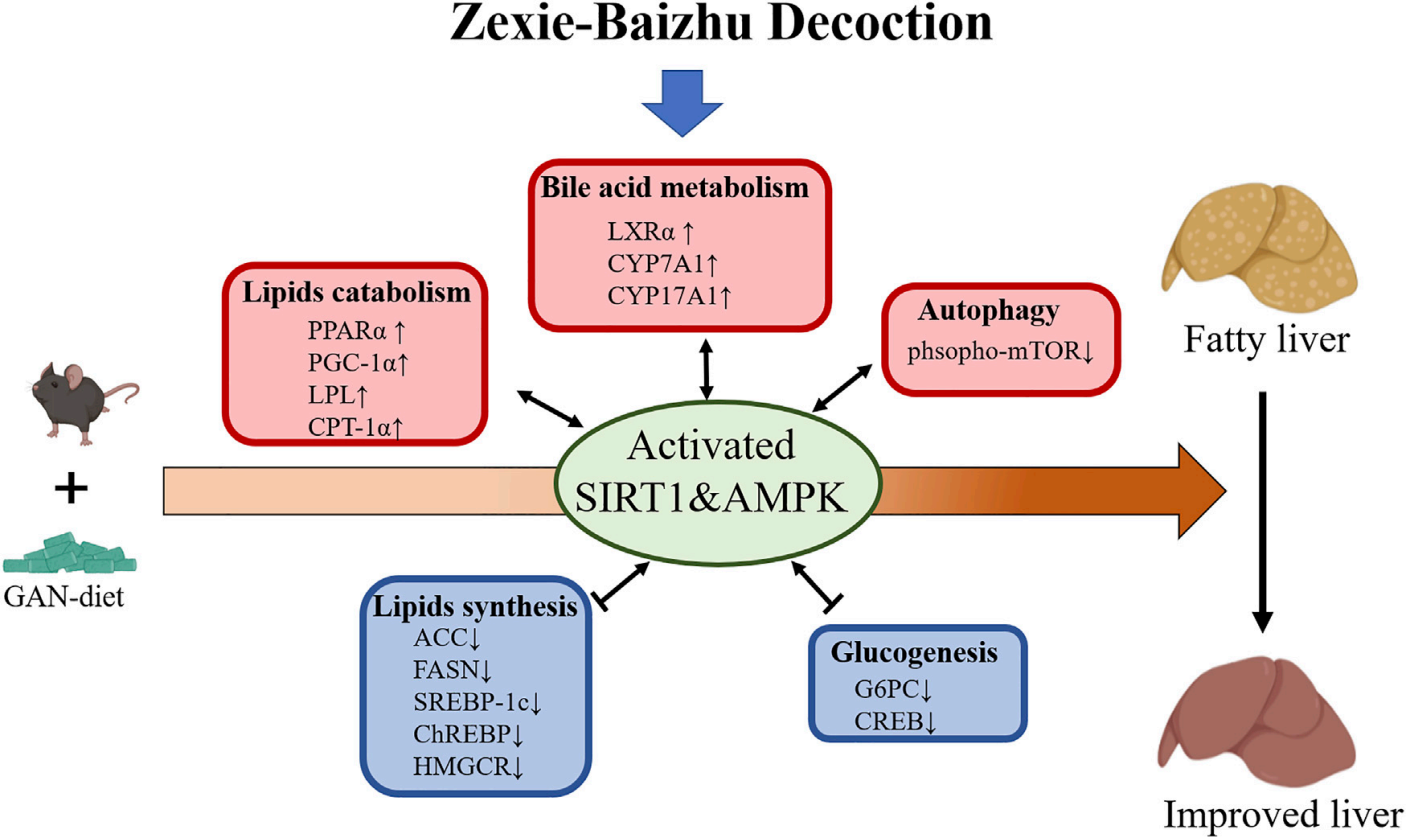
Proposed mechanism model for ZXBZ decoction treatment in NAFLD mice. GAN diet–induced lipid disorders in mice. ZXBZ decoction activated Sirt1 and AMPK, subsequently influenced related genes to improve lipid metabolism. The lipogenesis (SREBP-1c, ChREBP, ACC, FAS, and HMGCR) were suppressed, fatty utilization (PPARα, PGC-1α, LPL, CPT-1α) were activated, bile acid metabolism (LXRα, CYPs) were increased, gluconeogenesis (Creb, G6pc) was inhibited, and autophagy (mTOR) was prompted. As a result, the livers were protected from lipotoxicity.

In our study, ZXBZ decoction alleviated liver injury in the GAN diet–induced NAFLD model featured by lower ALT, AST, hepatosmatic index, and NAFLD activity score. Metabolomics hinted ZXBZ decoction promoted lipid utilization and transcriptomics implied that energy metabolisms, especially lipid metabolism, were improved, which was confirmed by the alleviated hepatic steatosis and dyslipidemia with ZXBZ decoction treatment *in vivo*. In metabolomics studies, two important potential biomarkers, L-carnitine and L-acetylcarnitine, were identified. Carnitine plays a key role in energy metabolism, transporting long-chain fatty acids into the mitochondria for oxidation and modulating the rise in the intramitochondrial acyl-CoA/CoA ratio (Tanphaichitr and Leelahagul, 1993). L-carnitine reduces fructose-mediated lipid accumulation by activating AMPK (Montesano et al., 2020), shown to be closely related to fatty acid β oxidation (Minokoshi et al., 2021). The mechanism of L-acetylcarnitine is also related to AMPK, inhibiting TNF-α-induced insulin resistance in skeletal muscle cells via the AMPK pathway (Zhang et al., 2009). Then, the interplays were uncovered in protein and mRNA levels. Two central energy sensors, Sirt1 and AMPK were activated, subsequently, lipogenesis-related proteins (SREBP-1c, ChREBP, ACC, FASN, and HMGCR) were suppressed, FAO involved genes (PPARα, PGC-1α, LPL, and CPT-1α) were activated, and lipid transport regulated genes (LXRα and CYPs) were increased (Figure 8).

As reported, AMPK and Sirt1 pathways are intertwined in mammalians, that is to say, AMPK and Sirt1 could activate and regulate each other, and they co-worked as an energy-sensing network sharing many common downstream targets (Ruderman et al., 2010; Cantó et al., 2009). AMPK can be activated in response to the change of the AMP/ATP ratio, and then the activated AMPK affects the NAD^+^/NADH ratio to activate Sirt1. At the same time, the activation of Sirt1 can also activate AMPK via LKB1 (Silvestre et al., 2014). However, the activation of AMPK and Sirt1 could also be independent. In neurons, the activation of AMPK needs LKB1 but does not require Sirt1 (Dasgupta and Milbrandt, 2007). In HepG2 cells, resveratrol could activate AMPK in Sirt1-dependent or Sirt1-independent manners (Hou et al., 2008; Shin et al., 2009). Also, in Sirt1^−/−^ mice, the activation of AMPK increased to rescue the depletion of Sirt1 (Pillai et al., 2010; Lee et al., 2008). In epithelial cells, the phosphorylation or inhibition of AMPK did not affect the activation of Sirt1 (Zu et al., 2010). Taken together, the regulation and interplay of these two key sensors of energy state in mammalian cells vary under different conditions. In the current study, we discovered that ZXBZ decoction could inhibit TG accumulation in both *in vivo* (Figure 5) and *in vitro* experiments (Supplementary Figure S5A). ZXBZ decoction increased AMPK phosphorylation significantly under the stimulation of 1 mM OA/PA for 24 h (Supplementary Figure S5B), while the Sirt1 level also increased slightly. Since the presence of OA would promote the expression of Sirt1 (Lim et al., 2013), PA without OA was used to stimulate HepG2 cells to further detect the effect of ZXBZ decoction on Sirt1. As shown in Supplementary Figure S5C, ZXBZ decoction elevated Sirt1 obviously. All these results indicated that ZXBZ decoction could regulate AMPK and Sirt1, which was also consistent with the result *in vivo*. Moreover, we also investigated the interplay of AMPK and Sirt1 activated by ZXBZ decoction through co-incubated with Compound C (specific AMPK inhibitor) and EX-527 (specific Sirt1 inhibitor). Interestingly, we discovered that ZXBZ decoction could activate AMPK and Sirt1 independently because the inhibition toward either AMPK or Sirt1 did not affect the activation of the other (Supplementary Figure S5D). It must be pointed out that TCM is characterized by multiple ingredients and targets; as a result, it was hard to determine the exact targets of ZXBZ decoction. Whether the activation of Sirt1 and AMPK was triggered by ZXBZ decoction directly, or derived from regulations of nuclear receptors, lipogenesis enzymes and lipid transporters, needs more experiments on transgenic mice.

As for glucose metabolism, ZXBZ decoction slightly reduced blood glucose, increased oral glucose tolerance, and decreased the expressions of glycogenesis-related genes (CREB and G6PC) along with the activation of Sirt1 and AMPK. Also, the inflammation was not severe in the 17-week GAN diet induction, since the severity of lobular inflammation (Figure 2G) was not serious, and the hepatic TNF-α and IL-6 were not increased significantly in the vehicle group (data not shown). The effects of ZXBZ decoction on glucose metabolism and inflammation need to be testified in another model.

Interestingly, the ZXBZ-L group exhibited lower hepatic enzyme levels but only slightly ameliorated hepatic lipid accumulation and dyslipidemia. There might be two reasons for this phenomenon: 1) the protective effects on hepatocytes might attribute to the increased level of PGC-1α, which also protected mitochondria from oxidative stress (Figure 6A). 2) Since the standard of steatosis is according to the overall lipid droplet areas, the ZXBZ-L and vehicle groups had similar ranges of steatosis (33–66%) (Figure 2F). But, the ZXBZ-L group had generally fewer large lipid droplets than the vehicle group (Figure 2F). Many previous studies demonstrated that the larger size of lipid droplets would make more serious hepatocyte injuries (Ferri et al., 2021; Mashek, 2021). Therefore, we speculated that the low dosage of ZXBZ decoction could protect livers by reducing the formation of large hepatic lipid droplets and increasing the PGC-1α expression.

Noteworthy, the characteristics of hyperglycemia and insulin resistance in NAFLD did not exacerbate in the 17-week GAN diet–induced mouse model. As a result, the possible improvements in glucose metabolism could not be observed in this model. The insulin resistance was not evident in this model (Supplementary Figures S4A,B), since the PI3K-Akt pathway was activated in the vehicle group as the feedback toward a high-fructose diet. The inhibition of GSK3β (S9 phosphorylation) was remarkable along with the activated Akt in the ZXBZ-L group (Supplementary Figure S4C). However, it was hard to get the conclusion that ZXBZ decoction could influence the PI3K/Akt pathway, as the activation was in three GAN diet groups compared to NC, which could be the feedback toward the GAN diet. Also, the transcription of G6PC was reduced in vehicle, ZXBZ-L, and H groups. It might be due to the fructose being the main sugar resource in the GAN diet, subsequently, leading to reduced gluconeogenesis in these groups. But ZXBZ groups still had significantly lower expressions of G6PC compared to vehicle, which also indicated the potential glucose metabolism regulatory ability of ZXBZ decoction. Also, whether ZXBZ decoction improved glucose metabolism needs further research based on STZ-induced or db/db mice.

Although there are several studies on the pharmacological effects of alcohol extracts of Zexie and Baizhu or monomers in the NAFLD treatment, the current study was focused on the initial-described and universal-used ZXBZ aqueous decoction for the first time. Moreover, the composition of active ingredients in aqueous decoction was first identified and discovered completely different compared to alcohol extracts. The combination of transcriptomics and metabolomics can demonstrate drug efficacies and mechanisms at multiple levels, especially fit in the research works of complex diseases and TCM with multiple ingredients and targets. Transcriptomics could reveal the expressions of related genes at overall levels, and metabolomics could reflect the holistic and real-time dynamic changes of endogenous metabolites after TCM intervention. The results emphasized the regulation of multiple targets and pathways and the whole energy sensing and regulatory network, highlighting the strength of TCM toward metabolic diseases. Of note, this study broadened the potential utilizations of ZXBZ decoction, helped understand the relationship between the mechanisms and functions of ZXBZ decoction and developed the natural herbs as complicated disease treatment candidates further.

## Data Availability

The original contributions presented in the study are publicly available. This data can be found here: https://submit.ncbi.nlm.nih.gov/subs/bioproject/SUB10918355/overview, BioProject ID PRJNA795724.
