# Supplementary material for: Multi-Omics Integration Analysis Identifies Lipid Disorder of a Non-Alcoholic Fatty Liver Disease (NAFLD) Mouse Model Improved by Zexie–Baizhu Decoction
Source: Front Pharmacol. 2022 Jun 20;13:858795. doi: 10.3389/fphar.2022.858795 (PMC9251488; doi:10.3389/fphar.2022.858795)
Supplement: Supplementary file 1 [file DataSheet1.DOCX]

**Supplementary Material**

Manuscript ID: 858795

**Contents**

[Methods 2](#_Toc100403896)

[1.Quantification of monosaccharides and oligosaccharides 2](#_Toc100403897)

[2.Quantification of nucleotides 2](#_Toc100403898)

[3. The representative total ion chromatogram of ZXBZ 3](#_Toc100403899)

[Supplementary Figures 4](#_Toc100403900)

[Supplementary Tables 10](#_Toc100403901)

# Methods

## 1.Quantification of monosaccharides and oligosaccharides

300 mg of ZXBZ powder was fixed to 25 mL water and centrifuged at 6000 rpm for 10 min. 15 mL of supernatant was added onto a treated solid-phase extraction column (6 mL of methanol, 10 mL of water pre-washed), and 10 mL of water was used for elution. The supernatant and the water eluate were collected and fixed to 50 mL. 0.5 mL of the solution was mixed with 0.5 mL of acetonitrile. The supernatant was removed and injected.

The chromatographic analysis was performed using a Thermo U3000 system coupled with a BEH Amide column (2.1×100 mm, 1.7 μm) and detected by a DAD detector. The column temperature was set as 60 ºC. The mobile phase was acetonitrile: water = 99:1 with 5 mmol/L ammonium acetate (A) and water with 20 mmol/L ammonium acetate (B). The gradient elution was performed as follows: 0-5 min, 95 % A; 5-25 min, 95% to 85% A; 25-30 min, 85% to 65% A; 30-35 min, 65%; 35-36 min, 65% to 95% A; 36-45 min,95% A. The injection volume was 2 μl, and the flow rate of the mobile phase was 0.35 mL/min.

## 2.Quantification of nucleotides

100 mg of ZXBZ powder was fixed to 25 mL of water. After being shaken and filtered, the supernatant was removed and injected.

The chromatographic analysis was performed using a Thermo U3000 system coupled with a Gemini C18 (50 mm*4.6 mm,3.5 μm) and detected by a UV detector. The column temperature was set as 30 ºC. The mobile phase was composed of acetonitrile (A) and water with 5 mmol/L ammonium acetate (B). The gradient elution was performed as follows: 0-5 min, 0 % A; 5-25 min, 0% to 10% A; 25-30 min, 10% to 95% A; 30-35 min, 95 %; 35-36 min, 95 % to 0 % A; 36-42 min, 0 % A. The injection volume was 15 μl, and the flow rate of the mobile phase was 0.8 mL/min.

## 3. The representative total ion chromatogram of ZXBZ

50 mg of ZXBZ powder was dissolved in 10 mL of 50% methanol-water. After being vortexed, sonicated for 30 min and centrifuged at 12000 rpm for 10 min. the supernatant was taken and injected into the sample.

The representative spectra of ZXBZ were analysed using an UPLC-ESI-MS system consisting of a Waters ACQUITY UPLC System (Waters Corp., Milford, MA, USA) coupled to a Xevo G2-S Q-TOF instrument (ACQUITY, WATERS, Milford, USA)

A Waters ACQUITY UPLC HSS T3 column (2.1 × 150 mm, 1.8 µm) was used for chromatographic separation maintained at 35 °C with 0.3 ml/min flow rate. The mobile phase consisted of 100% water (0.1 % formic acid) (A) and 100% acetonitrile (B). The linear gradient eluted from 0% B to 10% B (0-5 min), 10% B to 15% B (5-10 min), 15% B to 30% B (10-15 min), 30% B to 40% B (15-20 min), 40% B to 80% B (20-25 min), 80% B to 100% B (25-30 min), and 100% B to 0% B (35-40min). The autosampler was kept at 4 °C. A sample of 2 μL was injected.

Positive and negative ion mode with mass scan range of 100–1200 Da were conducted in these analyses. ESI source conditions were set as followings: capillary voltage, 2.5 kV (negative) and 3 kV (positive); cone voltage, 100 V; ion source temperature: 120 ℃; desolvation temperature: 500 ℃; cone gas flow rate: 30 L/h; desolvation gas flow rate: 1000 L/h. Leucine-enkephalin was used as the lock-mass reference compound both in positive mode and negative mode to ensure accuracy and reproducibility.

# Supplementary Figures


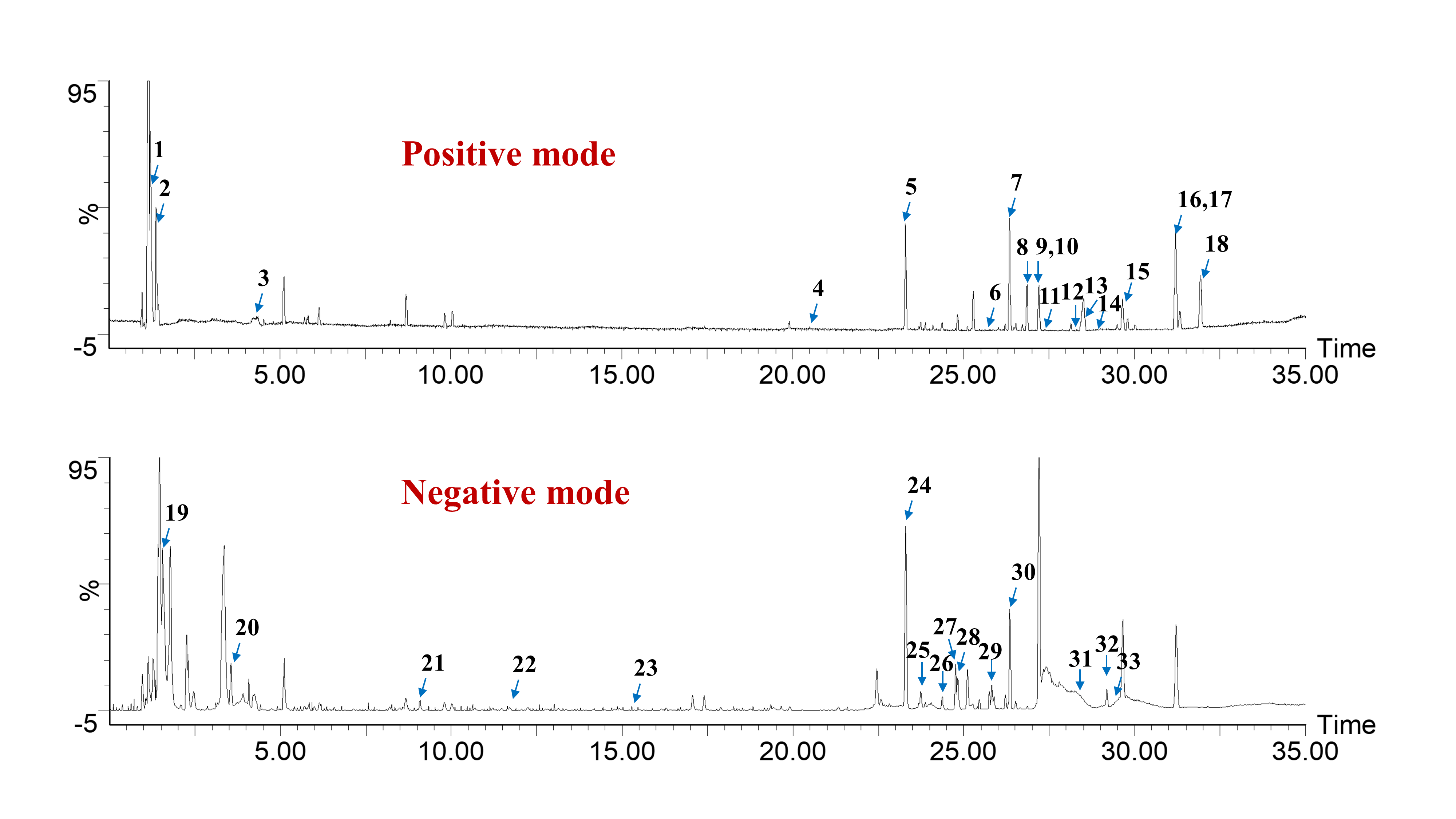


**Figure.S1** Representative total ion chromatogram of ZXBZ in positive and negative mode


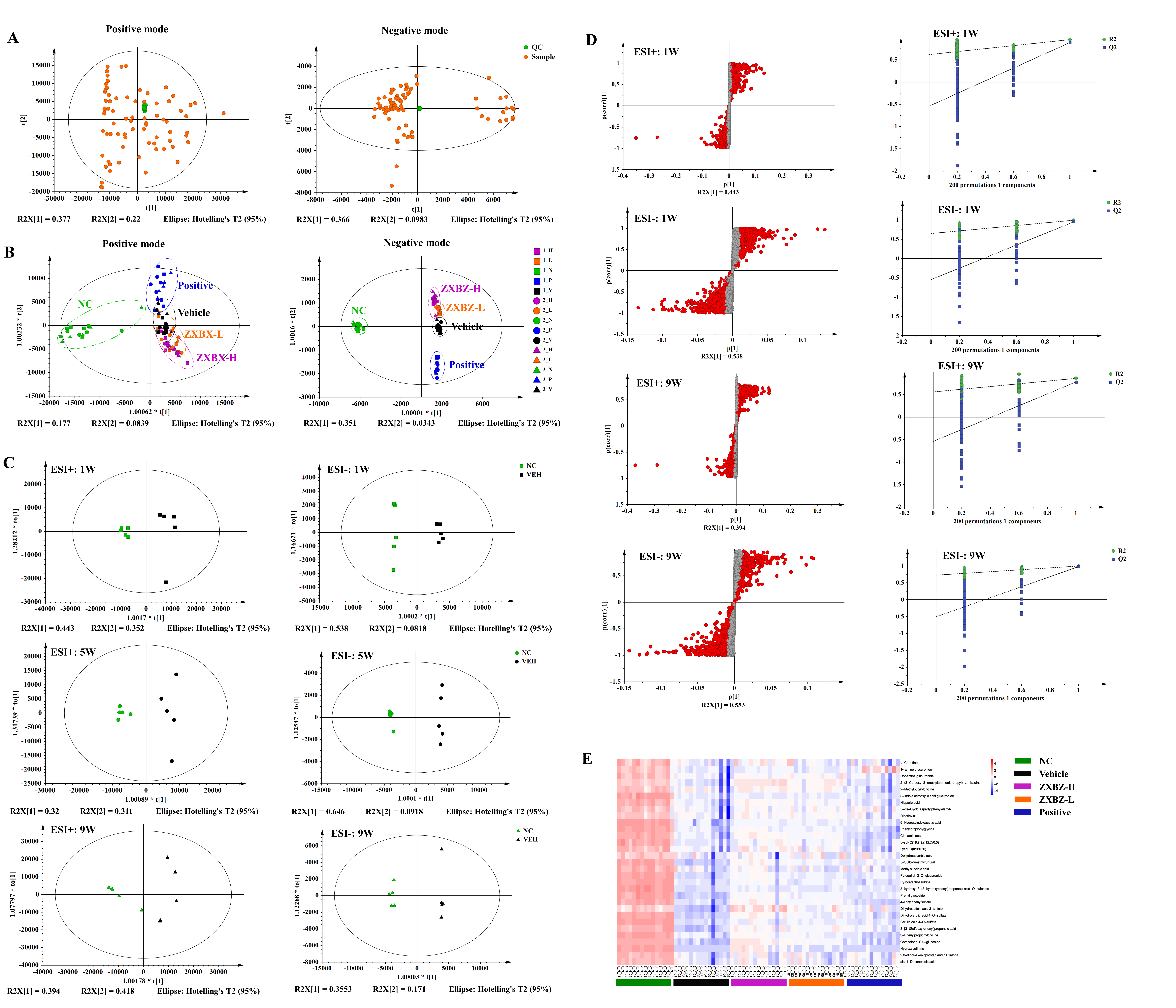


**Figure.S2** Biomarker search for Zexiebaizhu Decoction.

(A)PCA score plots of urine data collected in the positive mode and negative mode. (B) OPLS-DA score plot of urine data collected at different time points in the positive and negative ion mode. (C) O2PLS-DA score plot of urine data in the positive mode and negative mode at 1, 5, 9 weeks of drug administration. (D). OPLS-DA s-plot and permutations of urine data in the positive mode and negative mode at 1, 9 weeks. (E) Heatmap of 30 metabolites stably present in 3 samples.


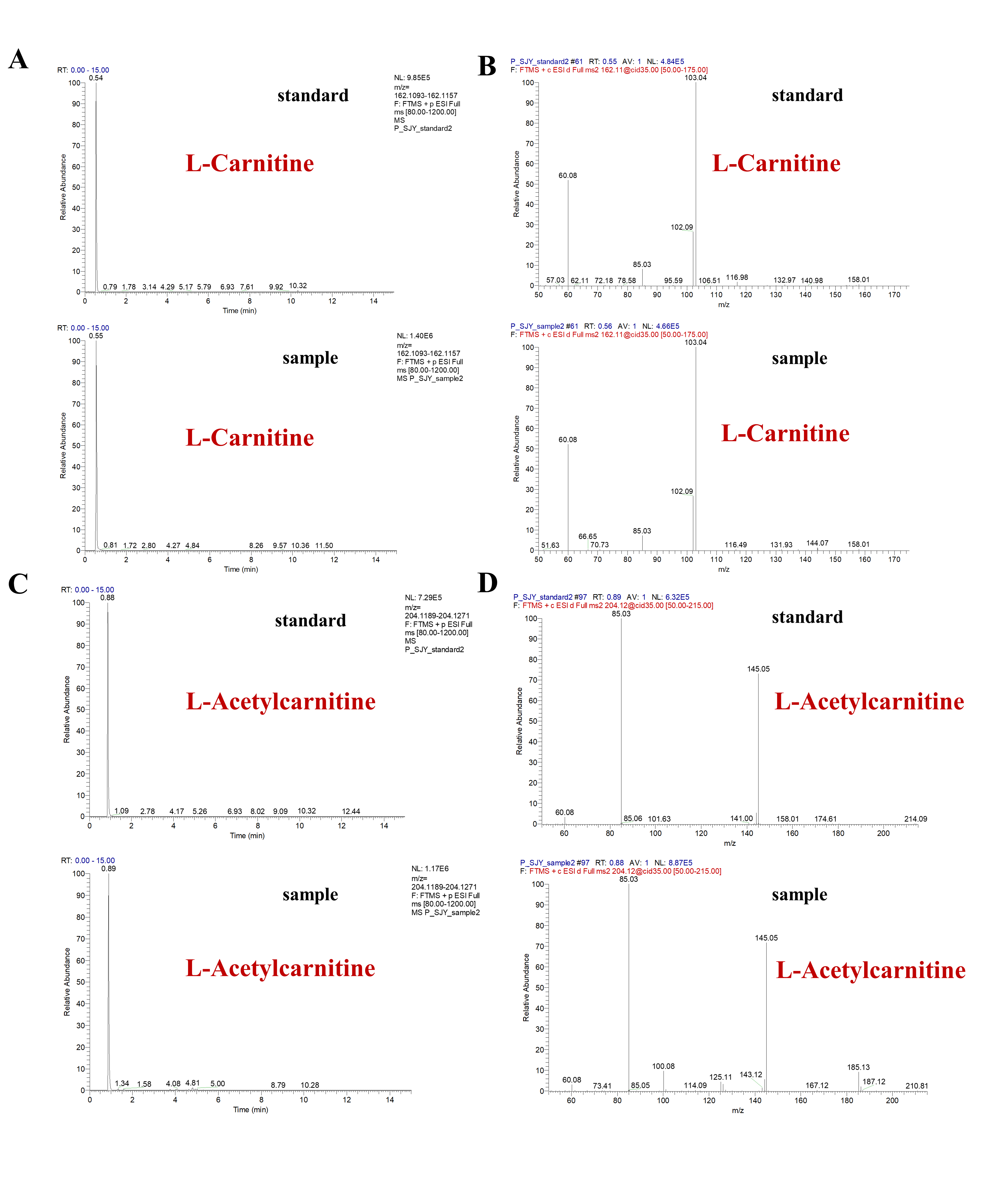


**Figure.S3** Confirmation of L-Carnitine and L-Acetylcarnitine. (A) MS spectra of L-Carnitine in standard and sample. (B) MS/MS spectra of L-Carnitine in standard and sample. (C) MS spectra of L-Acetylcarnitine in standard and sample. (D) MS/MS spectra of L-Acetylcarnitine in standard and sample.


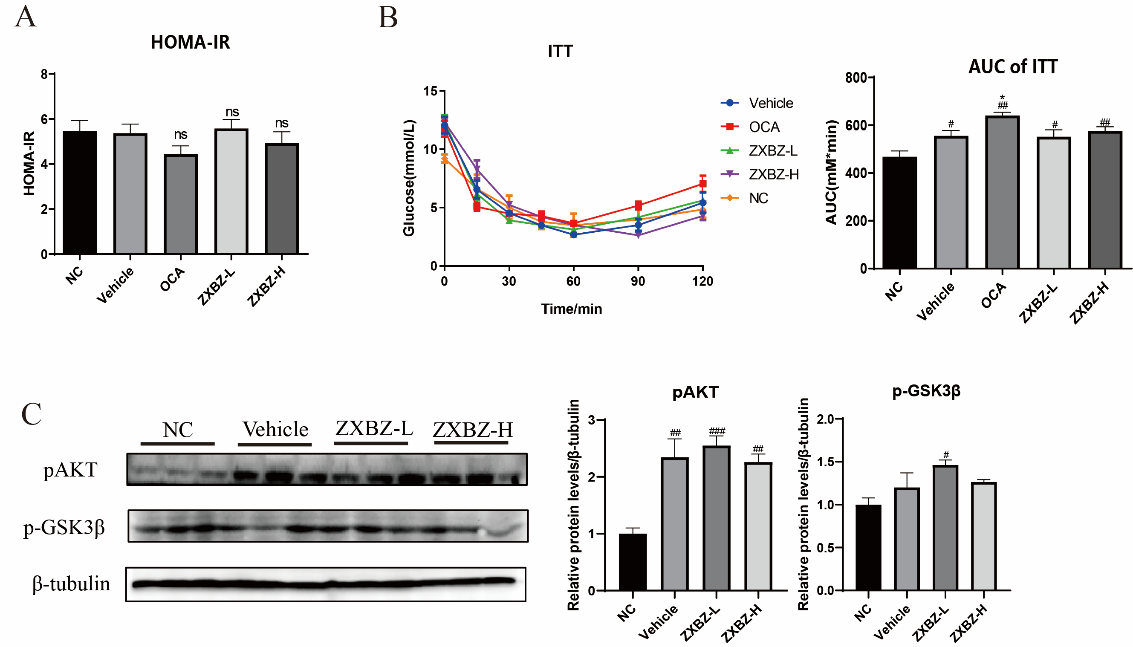


**Figure.S4 ZXBZ decoction slightly improved glucose metabolism and related genes.** (A) The HOMA-IR was performed at the 6th week of drug treatment. Data are the mean ± SEM (n =8-10 per group). (B) Insulin tolerance test assay and related area under the curve. Data are the mean ± SEM (n =8-10 per group). (C) The phospho-Akt and phospho-GSK3β normalized by β-tubulin. Data are mean ± SEM (n =3 per group). # p<0.05, ## p<0.01 compared to NC. * p<0.05, ** p<0.01 compared to Vehicle


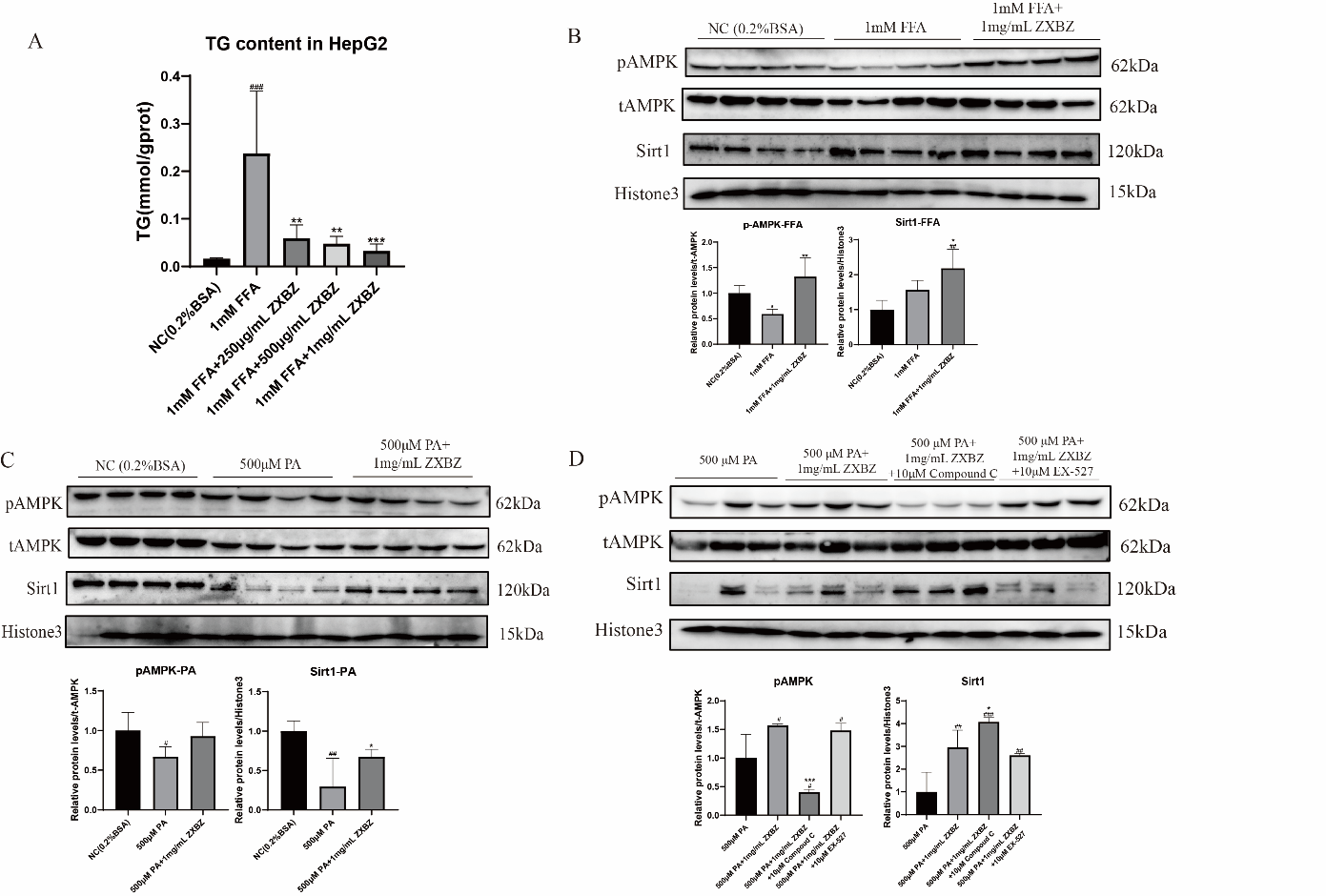


**Figure.S5** **ZXBZ decoction showed similar pharmacological effects on FFA or PA-induced HepG2 cells with the NAFLD mouse model.** (A)The TG contents in HepG2 cells treated with 0.2%BSA(NC), FFA (1 mM) or FFA (1 mM)/ZXBZ decoction. Data are mean ± SD (n =4 per group). ### p<0.001 compared to NC, * p<0.05, ** p<0.01, *** p<0.001 compared to FFA group. (B) The phospho-AMPK and Sirt1 levels in HepG2 cells treated with 0.2%BSA (NC), FFA (1 mM) or FFA (1 mM)/ZXBZ decoction (1 mg/mL). The data are the mean ± SD (n =4 per group). # p<0.05, ## p<0.01 compared to NC, * p<0.05, ** p<0.01 compared to FFA group. (C) The phospho-AMPK and Sirt1 levels in HepG2 cells treated with 0.2%BSA(NC), PA (500 μM) or PA (500 μM)/ZXBZ decoction (1 mg/mL). The data are the mean ± SD (n =4 per group). # p<0.05, ### p<0.001 compared to NC, * p<0.05, ** p<0.01 compared to PA group. (D) The phospho-AMPK and Sirt1 levels in HepG2 cells treated with PA (500 μM), PA (500 μM)/ZXBZ decoction (1 mg/mL), PA (500 μM)/ZXBZ decoction (1 mg/mL)/Compound C (10μM, pre-treated 1 hour) or PA (500 μM)/ZXBZ decoction (1 mg/mL)/EX-527 (10μM, pre-treated 1 hour). The data are the mean ± SD (n =3 per group). # p<0.05, ## p<0.01, ### p<0.001 compared to PA group, * p<0.05, ** p<0.01, *** p<0.001 compared to PA/ZXBZ group.

# Supplementary Tables

**TableS1. Characterization of chemical constituents in ZXBZ**

| Peak  number | RT  (min) | MS^1^ | Error  (ppm) | Fragment ions | Molecular formula | Identity |
| --- | --- | --- | --- | --- | --- | --- |
| 1 | 1.15 | 175.1194[M+H]^+^ | 2.3 | 130.0963,158.0912 | C_6_H_14_N_4_O_2_ | L-Arginin |
| 2 | 1.46 | 829.2825[M+H]^+^ | 0.7 | 163.0600,325.1129,343.1233,487.1654,685.2390 | C_30_H_52_O_26_ | verbascose |
| 3 | 4.26 | 182.0810[M+H]^+^ | -0.8 | 163.0600 | C_9_H_11_NO_3_ | (2S)-2-amino-3-(4-hydroxyphenyl)propanoic acid |
| 4 | 20.50 | 203.1792[-H_2_O+H]^+^ | -0.9 | 105.0693,119.0851,203.1773 | C_15_H_24_O | caryophyllene oxide |
| 5 | 23.30 | 505.3523[M+H]^+^ | -0.1 | 161.0953,215.1424,353.2467 | C_30_H_48_O_6_ | 16-oxoalisol A |
| 6 | 25.63 | 230.1538[M+H]^+^ | -0.4 | 160.0757,186.0920,214.1240 | C_15_H_19_NO | Atractylenolactam |
| 7 | 26.35 | 529.3521[M+H]^+^ | -0.6 | 215.1421,469.3310 | C_32_H_48_O_6_ | alisol Q 23-acetate |
| 8 | 26.83 | 305.2473[M+H]^+^ | -0.8 | 105.0695,233.1527 | C_20_H_32_O_2_ | oriediterpenol |
| 9 | 27.20 | 473.3627[M+H]^+^ | 0.3 | 145.1002,161.1314,201.1632,213.1626 | C_30_H_48_O_4_ | alisol G |
| 10 | 27.20 | 473.3624[-H_2_O+H]^+^ | -0.2 | 121.1009,145.1006,383.2941 | C_30_H_50_O_5_ | alisol A |
| 11 | 27.37 | 479.3495[M+Na]^+^ | -0.1 | 159.1166,201.1631,219.1733 | C_30_H_48_O_3_ | 11-deoxyalisol B |
| 12 | 28.41 | 545.3473[M+H]^+^ | 0 | 133.1017,161.1335,173.1336,369.2396 | C_32_H_48_O_7_ | alismalactone 23-acetate |
| 13 | 28.51 | 301.1411[M+Na]^+^ | 0.2 | 149.0228 | C_16_H_22_O_4_ | bis(2-methylpropyl) benzene-1,2-dicarboxylate |
| 14 | 28.84 | 495.3466[-H_2_O+H]^+^ | -0.5 | 157.1003,199.1472,495.3455 | C_32_H_48_O_5_ | alisol O |
| 15 | 29.65 | 469.3312[M+H]^+^ | -0.1 | 145.1007,383.2936 | C_30_H_44_O_4_ | alismanol E |
| 16 | 31.20 | 515.3732[-H_2_O+H]^+^ | 0.2 | 133.1006,515.3729 | C_32_H_52_O_6_ | alisol A 23-acetate |
| 17 | 31.21 | 515.3733[M+H]^+^ | 0.3 | 145.1007,173.1318,199.1475 | C_32_H_50_O_5_ | 25-anhydroalisol A 11-acetate |
| 18 | 31.94 | 265.2524[M+H]^+^ | -0.7 | 121.1069,135.1136 | C_18_H_32_O | (Z)-9,17-octadecadienal |
| 19 | 1.47 | 549.1668[M+HCOO]^-^ | -0.8 | 179.0564,191.0567341.1091 | C_18_H_32_O_16_ | raffinose |
| 20 | 3.51 | 711.2187[M+HCOO]^-^ | -2.0 | 179.0563,341.1082,485.1502 | C_24_H_42_O_21_ | D-stachyose |
| 21 | 9.10 | 493.2282[M+HCOO]^-^ | -1.7 | 285.1670,447.2220 | C_21_H_36_O_10_ | AtractylosideA |
| 22 | 11.71 | 193.0506[M-H]^-^ | -0.3 | 134.0369 | C_10_H_10_O_4_ | Ferulic acid |
| 23 | 15.21 | 161.0243[M-H_2_O-H]^-^ | -0.9 | 135.0445,179.0357 | C_9_H_8_O_4_ | Caffeic acid |
| 24 | 23.30 | 485.3273[M-H]^-^ | 0.2 | 279.1749,295.1700,367.2631,455.2799 | C_30_H_46_O_5_ | alisol C |
| 25 | 23.74 | 527.3373[M-H]^-^ | -0.9 | 397.2317,467.3159 | C_32_H_48_O_6_ | alisol C 23-acetate |
| 26 | 24.38 | 467.3159[M-H]^-^ | -1.7 | 325.1821,367.2633 | C_30_H_44_O_4_ | alismanol B |
| 27 | 24.76 | 551.3582[M+HCOO]^-^ | -1.3 | 233.1548,341.2125 | C_30_H_50_O_6_ | 13β,17β-epoxyalisol A |
| 28 | 24.82 | 531.3321[M+HCOO]^-^ | -1.1 | 295.1693,379.2248,409.2731 | C_30_H_46_O_5_ | 16-oxo-11-anhydroalisol A |
| 29 | 25.83 | 519.3321[M-H]^-^ | -1.1 | 325.2520,475.3405 | C_30_H_48_O_7_ | alisol P |
| 30 | 26.35 | 509.3274[M-H]^-^ | 0.3 | 295.1736,321.1857,361.2190,419.2577,449.3097 | C_32_H_46_O_5_ | alisol L 23-acetate |
| 31 | 28.42 | 543.3326[M-H]^-^ | -0.3 | 303.1964,417.2636 | C_32_H_48_O_7_ | alisol M 23-acetate |
| 32 | 29.19 | 517.3527[M+HCOO]^-^ | -1.4 | 299.2015 | C_30_H_48_O_4_ | alisol B |
| 33 | 29.37 | 549.3800[M+HCOO]^-^ | 0.5 | 351.2664,397.2740 | C_31_H_52_O_5_ | 23-O-methylalisol A |

**Table.S2 The detailed identification information and shifting trends of biomarkers**

|  | NO. | HMDB ID | Rt | Metabolites | Formula | Monoisotopic Mass | Adduct | Adduct m/z | Delta (ppm) | N-1W vs. V-1W | | | | N-5W vs. V-5W | | | | N-9W vs. V-9W | | | |
| --- | --- | --- | --- | --- | --- | --- | --- | --- | --- | --- | --- | --- | --- | --- | --- | --- | --- | --- | --- | --- | --- |
|  |  |  |  |  |  |  |  |  |  | V/N | H/V | L/V | P/V | V/N | H/V | L/V | P/V | V/N | H/V | L/V | P/V |
| ESI+ | 1 | HMDB0252494 | 0.54 | Fructose-lysine | C_12_H_24_N_2_O_7_ | 308.1584 | M+H | 309.1656 | 1 | ↓^##^ | ↑ | ↓ | ↓ | — | — | — | — | — | — | — | — |
|  | 2 | HMDB0000062 | 0.57 | *L*-Carnitine | C_7_H_15_NO_3_ | 161.1052 | M+H | 162.1125 | 2 | ↓^#^ | ↑ | ↑ | ↓ | ↓^#^ | ↑* | ↑ | ↑ | ↓^#^ | ↑* | ↑ | ↑ |
|  | 3 | HMDB0003553 | 0.84 | Stachyose | C_24_H_42_O_21_ | 666.2219 | M+K | 705.1850 | 1 | ↓^#^ | ↑ | ↓ | ↓ | ↓^##^ | ↑* | ↑** | ↑ | — | — | — | — |
|  | 4 | HMDB0000201 | 1.10 | *L*-Acetylcarnitine | C_9_H_17_NO_4_ | 203.1158 | M+H | 204.1230 | 0 | ↓^###^ | ↓ | ↓ | ↓ | — | — | — | — | ↓^#^ | ↑ | ↑ | ↑ |
|  | 5 | HMDB0028867 | 1.16 | Hydroxyprolyl-Leucine | C_11_H_20_N_2_O_4_ | 244.1423 | M+H | 245.1496 | 1 | ↓^###^ | ↓ | ↑ | ↓ | ↓^#^ | ↑ | ↑ | ↓ | — | — | — | — |
|  | 6 | HMDB0010328 | 1.31 | Tyramine glucuronide | C_14_H_19_NO_7_ | 313.1162 | M+H | 314.1234 | 1 | ↓^#^ | ↓ | ↑ | ↑ | ↓^#^ | ↑ | ↑ | ↑ | ↓^#^ | ↑ | ↑ | ↑ |
|  | 7 | HMDB0000014 | 1.54 | Deoxycytidine | C_9_H_13_N_3_O_4_ | 227.0906 | M+H | 228.0979 | 0 | — | — | — | — | ↓^##^ | ↑**** | ↑** | ↑ | — | — | — | — |
|  | 8 | HMDB0010329 | 1.62 | Dopamine glucuronide | C_14_H_19_NO_8_ | 329.1111 | M+H | 330.1183 | 1 | ↓^#^ | ↓ | ↑ | ↓ | ↓^##^ | ↑ | ↑ | ↑ | ↓^#^ | ↑ | ↑ | ↑ |
|  | 9 | HMDB0003282 | 1.66 | 1-Methylguanine | C_6_H_7_N_5_O | 165.0651 | M+H | 166.0723 | 1 | ↓^#^ | ↑ | ↓ | ↓ | — | — | — | — | — | — | — | — |
|  | 10 | HMDB0029443 | 1.92 | *L*-Pyridosine | C_12_H_18_N_2_O_4_ | 254.1267 | M+H | 255.1339 | 1 | ↓^#^ | ↑ | — | — | ↓^##^ | — | ↑ | — | — | — | — | — |
|  | 11 | HMDB0011654 | 2.74 | 2-(3-Carboxy-3-(methylammonio)propyl)-L-histidine | C_11_H_19_N_4_O_4_ | 271.1406 | M+H | 271.1406 | 3 | ↓^##^ | ↑* | ↑ | ↓ | ↓^##^ | ↑*** | ↑**** | ↑ | ↓^#^ | ↑*** | ↑**** | ↑ |
|  | 12 | HMDB0004193 | 3.52 | N1-Methyl-2-pyridone-5-carboxamide | C_7_H_8_N_2_O_2_ | 152.0586 | M+H | 153.0659 | 0 | ↓^##^ | ↑ | ↓ | ↓ | — | — | — | — | — | — | — | — |
|  | 13 | HMDB0011175 | 3.65 | Leucylproline | C_11_H_20_N_2_O_3_ | 228.1474 | M+H | 229.1547 | 1 | ↓^###^ | ↓ | ↓ | ↓ | — | — | — | — | — | — | — | — |
|  | 14 | HMDB0060017 | 3.98 | Pyrogallol-2-*O*-glucuronide | C_12_H_14_O_9_ | 302.0638 | M+H | 303.0711 | 1 | ↓^#^ | ↑* | ↑ | ↑ | ↓^##^ | ↑*** | ↑*** | ↑ | — | — | — | — |
|  | 15 | HMDB0000210 | 4.14 | Pantothenic acid | C_9_H_17_NO_5_ | 219.1107 | M+H | 220.1179 | 0 | — | — | — | — | ↓^#^ | ↑ | ↑ | ↑ | ↓^#^ | ↑* | ↑ | ↑ |
|  | 16 | HMDB0059997 | 4.64 | Dihyroxy-1H-indole glucuronide I | C_14_H_15_NO_8_ | 325.0798 | M+H | 326.0870 | 0 | ↓^###^ | ↓ | ↓ | ↓**** | — | — | — | — | ↓^#^ | ↑* | ↑* | ↓ |
|  | 17 | HMDB0000339 | 4.77 | 2-Methylbutyrylglycine | C_7_H_13_NO_3_ | 159.0895 | M+H | 160.0968 | 0 | ↓^####^ | ↑ | ↓ | ↓* | ↓^#^ | ↑ | ↑ | ↓* | ↓^#^ | ↑* | ↑* | ↓ |
|  | 18 | HMDB0013189 | 4.83 | 3-Indole carboxylic acid glucuronide | C_15_H_15_NO_8_ | 337.0798 | M+H | 338.0870 | 1 | ↓^#^ | ↑ | ↑ | ↓ | ↓^##^ | ↓ | ↑ | ↓ | ↓^#^ | ↑ | ↑ | ↓ |
|  | 19 | HMDB0000714 | 4.95 | Hippuric acid | C_9_H_9_NO_3_ | 179.0582 | M+H | 180.0655 | 0 | ↓^###^ | ↑* | ↑ | ↑ | ↓^##^ | ↑*** | ↑** | ↑ | ↓^#^ | ↑*** | ↑** | ↑ |
|  | 20 | HMDB0031360 | 4.97 | *L*-cis-Cyclo(aspartylphenylalanyl) | C_13_H_14_N_2_O_4_ | 262.0954 | M+H | 263.1026 | 0 | ↓^#^ | ↑ | ↑ | ↑ | ↓^##^ | ↑ | ↑ | ↑ | ↓^#^ | ↑* | ↑** | ↑ |
|  | 21 | HMDB0000244 | 5.04 | Riboflavin | C_17_H_20_N_4_O_6_ | 376.1383 | M+H | 377.1456 | 0 | ↓^###^ | ↑ | ↑ | ↓ | ↓^#^ | ↑ | ↑ | ↓ | ↓^#^ | ↑ | ↑* | ↑ |
|  | 22 | HMDB0000763 | 5.30 | 5-Hydroxyindoleacetic acid | C_10_H_9_NO_3_ | 191.0582 | M+H | 192.0655 | 0 | ↓^###^ | ↑ | ↑ | ↓ | ↓^##^ | ↑** | ↑ | ↑ | ↓^##^ | ↑* | ↑** | ↑ |
|  | 23 | HMDB0114743 | 5.33 | LysoPA(18:3(6Z,9Z,12Z)/0:0) | C_21_H_37_O_7_P | 432.2277 | M+H | 433.2350 | 4 | ↓^##^ | ↑ | ↓ | ↓ | — | — | — | — | — | — | — | — |
|  | 24 | HMDB0094728 | 5.56 | 2-Hepteneoylglycine | C_9_H_15_NO_3_ | 185.1052 | M+H | 186.1125 | 1 | — | — | — | — | ↓^#^ | ↑*** | ↑*** | ↑ | — | — | — | — |
|  | 25 | HMDB0061189 | 5.59 | 3-Hydroxyisovalerylcarnitine | C_12_H_23_NO_5_ | 261.1576 | M+H-2H_2_O | 226.1449 | 5 | — | — | — | — | — | — | — | — | ↓^#^ | ↑ | ↑ | ↑ |
|  | 26 | HMDB0013809 | 5.68 | (*E*)-2-octenal | C_8_H_14_O | 126.1045 | M+H | 127.1117 | 1 | — | — | — | — | ↓^#^ | ↑* | ↑ | ↓ | ↓^#^ | ↑* | ↑ | ↑ |
|  | 27 | HMDB0000860 | 5.86 | Phenylpropionylglycine | C_11_H_13_NO_3_ | 207.0895 | M+H | 208.0968 | 0 | ↓^##^ | ↑ | ↑ | ↓ | ↓^##^ | ↑* | ↑ | ↓ | ↓^#^ | ↑* | ↑** | ↑ |
|  | 28 | HMDB0000567 | 6.05 | Cinnamic acid | C_9_H_8_O_2_ | 148.0524 | M+H-H_2_O | 131.0497 | 6 | ↓^###^ | ↑* | ↑ | ↓* | ↓^##^ | ↑** | ↑* | ↓** | ↓^##^ | ↑**** | ↑** | ↑ |
|  | 29 | HMDB0010404 | 8.94 | LysoPC(22:6(4Z,7Z,10Z,13Z,16Z,19Z)/0:0) | C_30_H_50_NO_7_P | 567.3325 | M+H | 568.3398 | 2 | ↓^#^ | ↑ | ↑ | ↓ | — | — | — | — | — | — | — | — |
|  | 30 | HMDB0010386 | 8.97 | LysoPC(18:2(9Z,12Z)/0:0) | C_26_H_50_NO_7_P | 519.3325 | M+H | 520.3398 | 1 | ↓^#^ | ↑ | ↑ | ↓ | ↓^##^ | ↑ | ↑* | ↓* | ↓^#^ | ↑* | ↑* | ↑ |
|  | 31 | HMDB0240262 | 9.25 | LysoPC(0:0/16:0) | C_24_H_50_NO_7_P | 495.3325 | M+H | 496.3398 | 1 | ↓^##^ | ↑ | ↑ | ↓ | ↓^##^ | ↑ | ↑ | ↓* | ↓^#^ | ↑* | ↑ | ↑ |
| ESI- | 32 | HMDB0060649 | 0.84 | Ascorbic acid 2-sulfate | C_6_H_8_O_9_S | 255.9889 | M-H | 254.9816 | 0 | — | — | — | — | ↑^##^ | ↓ | ↓ | ↓ | — | — | — | — |
|  | 33 | HMDB0000428 | 1.12 | 3-Hydroxyglutaric acid | C_5_H_8_O_5_ | 148.0372 | M-H | 147.0299 | 1 | — | — | — | — | — | — | — | — | ↓^###^ | ↑ | ↓ | ↑ |
|  | 34 | HMDB0001264 | 1.51 | Dehydroascorbic acid | C_6_H_6_O_6_ | 174.0164 | M-H | 173.0092 | 0 | ↓^##^ | ↑ | ↑ | ↑ | ↓^#^ | ↑ | ↑ | ↑ | ↓^###^ | ↑ | ↑ | ↑ |
|  | 35 | HMDB0059752 | 3.03 | 5-Sulfoxymethylfurfural | C_6_H_6_O_6_S | 205.9885 | M-H | 204.9812 | 1 | ↓^#^ | ↑** | ↑** | ↑* | ↓^###^ | ↑* | ↑**** | ↑ | ↓^##^ | ↑* | ↑ | ↑ |
|  | 36 | HMDB0001844 | 3.44 | Methylsuccinic acid | C_5_H_8_O_4_ | 132.0423 | M-H | 131.0350 | 0 | ↓^##^ | ↓ | ↓ | ↑ | ↓^##^ | ↑ | ↓ | ↑ | ↓^##^ | ↑ | ↑ | ↑ |
|  | 37 | HMDB0000195 | 3.68 | Inosine | C_10_H_12_N_4_O_5_ | 268.0808 | M-H | 267.0735 | 0 | ↑^#^ | ↓ | ↑ | ↓ | ↑^#^ | ↑ | ↑ | ↑ | — | — | — | — |
|  | 38 | HMDB0001563 | 3.93 | 1-Methylguanosine | C_11_H_15_N_5_O_5_ | 297.1073 | M-H | 296.1000 | 0 | ↑^###^ | ↓* | ↓ | ↑ | — | — | — | — | — | — | — | — |
|  | 39 | HMDB0060017 | 4.03 | Pyrogallol-2-*O*-glucuronide | C_12_H_14_O_9_ | 302.0638 | M-H | 301.0565 | 0 | ↓^##^ | ↑** | ↑** | ↑ | ↓^###^ | ↑*** | ↑**** | ↑** | ↓^##^ | ↑* | ↑* | ↑* |
|  | 40 | HMDB0059724 | 4.11 | Pyrocatechol sulfate | C_6_H_6_O_5_S | 189.9936 | M-H | 188.9863 | 0 | ↓^##^ | ↑** | ↑ | ↓ | ↓^####^ | ↑** | ↑** | ↓ | ↓^##^ | ↑ | ↑* | ↑ |
|  | 41 | HMDB0059967 | 4.38 | 3-hydroxy-3-(3-hydroxyphenyl)propanoic acid-*O*-sulphate | C_9_H_10_O_7_S | 262.0147 | M-H | 261.0074 | 0 | ↓^##^ | ↑* | ↑** | ↑* | ↓^####^ | ↑* | ↑ | ↑ | ↓^##^ | ↑ | ↑ | ↑ |
|  | 42 | HMDB0013130 | 4.75 | Glutarylcarnitine | C_12_H_21_NO_6_ | 275.1369 | M-H_2_O-H | 256.1185 | 2 | ↑^####^ | ↓* | ↓ | ↓ | ↑^###^ | ↓ | ↓ | ↑ | ↑^#^ | ↓ | ↑ | ↑ |
|  | 43 | HMDB0031876 | 4.77 | Prenyl glucoside | C_11_H_20_O_6_ | 248.1260 | M+FA-H | 293.1242 | 0 | ↓^#^ | ↑** | ↑* | ↑* | ↓^###^ | ↑** | ↑*** | ↑ | ↓^##^ | ↑* | ↑** | ↑* |
|  | 44 | HMDB0062551 | 4.78 | 4-Ethylphenylsulfate | C_8_H_10_O_4_S | 202.0300 | M+FA-H | 247.0282 | 0 | ↓^##^ | ↑** | ↑*** | ↑ | ↓^###^ | ↑* | ↑** | ↓* | ↓^##^ | ↑ | ↑* | ↓ |
|  | 45 | HMDB0041721 | 4.79 | Dihydrocaffeic acid 3-sulfate | C_9_H_10_O_7_S | 262.0147 | M-H | 261.0074 | 0 | ↓^#^ | ↑* | ↑** | ↑* | ↓^####^ | ↑** | ↑** | ↑ | ↓^##^ | ↑* | ↑ | ↑ |
|  | 46 | HMDB0041724 | 4.91 | Dihydroferulic acid 4-*O*-sulfate | C_10_H_12_O_7_S | 276.0304 | M-H | 275.0231 | 1 | ↓^##^ | ↑**** | ↑**** | ↑ | ↓^###^ | ↑** | ↑** | ↑ | ↓^##^ | ↑* | ↑ | ↑ |
|  | 47 | HMDB0029200 | 4.99 | Ferulic acid 4-*O*-sulfate | C_10_H_10_O_7_S | 274.0147 | M-H | 273.0074 | 0 | ↓^##^ | ↑ | ↑* | ↑ | ↓^####^ | ↑ | ↑* | ↑ | ↓^##^ | ↑ | ↑ | ↑ |
|  | 48 | HMDB0094710 | 5.01 | 3-[3-(Sulfooxy)phenyl]propanoic acid | C_9_H_10_O_6_S | 246.0198 | M-H | 245.0125 | 0 | ↓^##^ | ↑*** | ↑*** | ↑** | ↓^####^ | ↑*** | ↑**** | ↑* | ↓^##^ | ↑* | ↑* | ↑ |
|  | 49 | HMDB0000821 | 5.27 | Phenylacetylglycine | C_10_H_11_NO_3_ | 193.0739 | M-H | 192.0666 | 1 | ↑^#^ | ↓ | ↓ | ↓ | ↑^###^ | ↓ | ↓ | ↓ | — | — | — | — |
|  | 50 | HMDB0011686 | 5.43 | p-Cresol glucuronide | C_13_H_16_O_7_ | 284.0896 | 2M-H | 567.1719 | 1 | ↑^####^ | ↓ | ↑ | ↓ | ↑^####^ | ↓ | ↓* | ↓* | — | — | — | — |
|  | 51 | HMDB0000424 | 5.70 | 2-Hydroxydecanedioic acid | C_10_H_18_O_5_ | 218.1154 | M-H | 217.1081 | 0 | — | — | — | — | ↓^##^ | ↑ | ↑ | ↓ | ↓^##^ | ↑ | ↑ | ↑ |
|  | 52 | HMDB0000888 | 5.73 | Undecanedioic acid | C_11_H_20_O_4_ | 216.1362 | M-H | 215.1289 | 1 | — | — | — | — | — | — | — | — | ↓^#^ | ↑ | ↑ | ↑ |
|  | 53 | HMDB0000671 | 5.82 | Indolelactic acid | C_11_H_11_NO_3_ | 205.0739 | M-H | 204.0666 | 0 | — | — | — | — | — | — | — | — | ↓^#^ | ↑ | ↑ | ↑ |
|  | 54 | HMDB0062445 | 5.85 | alpha-CEHC glucuronide | C_22_H_30_O_10_ | 454.1839 | M-H | 453.1766 | 0 | ↑^####^ | ↑ | ↑ | ↓*** | ↑^###^ | ↑ | ↑ | ↓ | ↑^#^ | ↓ | ↑ | ↓ |
|  | 55 | HMDB0002042 | 5.89 | 3-Phenylpropionylglycine | C_11_H_13_NO_3_ | 207.0895 | M-H | 206.0823 | 0 | ↓^##^ | ↑**** | ↑ | ↓* | ↓^###^ | ↑* | ↑ | ↓** | ↓^###^ | ↑* | ↑* | ↑ |
|  | 56 | HMDB0029772 | 6.14 | Corchoionol C 9-glucoside | C_19_H_30_O_8_ | 386.1941 | M-H | 385.1868 | 0 | ↓^###^ | ↑**** | ↑** | ↑ | ↓^##^ | ↑** | ↑*** | ↑ | ↓^##^ | ↑* | ↑** | ↑ |
|  | 57 | HMDB0001390 | 6.29 | Hydroxycotinine | C_10_H_12_N_2_O_2_ | 192.0899 | 2M-H | 383.1725 | 4 | ↓^##^ | ↑** | ↑ | — | ↓^###^ | ↑** | ↑ | ↑ | ↓^##^ | ↑ | ↑ | ↑ |
|  | 58 | HMDB0062618 | 6.61 | 2,3-dinor-6-oxoprostaglandin F1alpha | C_18_H_30_O_6_ | 342.2042 | M+FA-H | 387.2024 | 1 | ↓^##^ | ↑ | ↑ | ↑ | ↓^###^ | ↑ | ↑ | ↑ | ↓^###^ | ↑ | ↑ | ↑ |
|  | 59 | HMDB0002366 | 7.03 | Tiglylcarnitine | C_12_H_21_NO_4_ | 243.1471 | M-H_2_O-H | 224.1287 | 2 | ↑^####^ | ↑ | ↑ | ↓ | ↑^###^ | ↑ | ↑ | ↓ | ↑^#^ | ↓ | ↑ | ↑ |
|  | 60 | HMDB0000603 | 7.29 | cis-4-Decenedioic acid | C_10_H_16_O_4_ | 200.1049 | 2M-H | 399.2024 | 1 | ↓^##^ | ↑ | ↑ | ↑ | ↓^###^ | ↑ | ↑ | ↓ | ↓^####^ | ↑ | ↑ | ↑ |
